# Low Ciprofloxacin Concentrations Select Multidrug-Resistant Mutants Overproducing Efflux Pumps in Clinical Isolates of Pseudomonas aeruginosa

**DOI:** 10.1128/spectrum.00723-22

**Published:** 2022-08-24

**Authors:** Fernando Sanz-García, Sara Hernando-Amado, Carla López-Causapé, Antonio Oliver, José Luis Martínez

**Affiliations:** a Centro Nacional de Biotecnología, CSIC, Madrid, Spain; b Departamento de Microbiología, Medicina Preventiva y Salud Pública, Universidad de Zaragoza, Zaragoza, Spain; c Servicio de Microbiología Hospital Universitario Son Espases, IdISBa, CIBERINFEC, Palma de Mallorca, Spain; Emory University School of Medicine

**Keywords:** *Pseudomonas aeruginosa*, sub-MIC selective window, One-Health, clinical isolates, MDR efflux pumps, ciprofloxacin, cross-resistance

## Abstract

Low antibiotic concentrations present in natural environments are a severe and often neglected threat to public health. Even if they are present below their MICs, they may select for antibiotic-resistant pathogens. Notably, the minimal subinhibitory concentrations that select resistant bacteria, and define the respective sub-MIC selective windows, differ between antibiotics. The establishment of these selective concentrations is needed for risk-assessment studies regarding the presence of antibiotics in different habitats. Using short-term evolution experiments in a set of 12 Pseudomonas aeruginosa clinical isolates (including high-risk clones with ubiquitous distribution), we have determined that ciprofloxacin sub-MIC selective windows are strain specific and resistome dependent. Nonetheless, in all cases, clinically relevant multidrug-resistant (MDR) mutants emerged upon exposure to low ciprofloxacin concentrations, with these concentrations being below the levels reported in ciprofloxacin-polluted natural habitats where P. aeruginosa can be present. This feature expands the conditions and habitats where clinically relevant quinolone-resistant mutants can emerge. In addition, we established the lowest concentration threshold beyond which P. aeruginosa, regardless of the strain, becomes resistant to ciprofloxacin. Three days of exposure under this sub-MIC “risk concentration” led to the selection of MDR mutants that displayed resistance mechanisms usually ascribed to high selective pressures, i.e., the overproduction of the efflux pumps MexCD-OprJ and MexEF-OprN. From a One-Health viewpoint, these data stress the transcendent role of low drug concentrations, which can be encountered in natural ecosystems, in aggravating the antibiotic resistance problem, especially when it comes to pathogens of environmental origin.

**IMPORTANCE** It has been established that antibiotic concentrations below MICs can select antibiotic-resistant pathogens, a feature of relevance for analyzing the role of nonclinical ecosystems in antibiotic resistance evolution. The range of concentrations where this selection occurs defines the sub-MIC selective window, whose width depends on the antibiotic. Herein, we have determined the ciprofloxacin sub-MIC selective windows of a set of Pseudomonas aeruginosa clinical isolates (including high-risk clones with worldwide distribution) and established the lowest concentration threshold, notably an amount reported to be present in natural ecosystems, beyond which this pathogen acquires resistance. Importantly, our results show that this ciprofloxacin sub-MIC selects for multidrug-resistant mutants overproducing clinically relevant efflux pumps. From a One-Health angle, this information supports that low antimicrobial concentrations, present in natural environments, may have a relevant role in worsening the antibiotic resistance crisis, particularly regarding pathogens with environmental niches, such as P. aeruginosa.

## INTRODUCTION

Antibiotics represent one of humankind’s most significant medical discoveries. Unfortunately, during the last decades, the emergence and spread of antibiotic resistance (AR) have hindered the efficacy of antibiotics. Consequently, AR is currently considered as a major threat to public health worldwide (1). The emergence of clinically relevant antimicrobial resistance has been traditionally associated with exposure to high therapeutic concentrations of antibiotics. However, it has been stated that low antimicrobials levels, below the MIC, might also contribute to the current AR crisis, since they may select antibiotic-resistant mutants (2, 3). Besides, they may alter other elements of bacterial physiology with relevance for AR evolution such as mutation rate, recombination, or cell-to-cell interactions (4–6). Notably, these low antibiotics concentrations are found in several different environments, including natural ecosystems (7). Consequently, defining the sub-MIC ranges of antibiotics of clinical importance that select for resistant mutants and establishing the concentration threshold above which drug pollution poses a risk are of ultimate importance. This range, which depends on the bacterial species and antibiotic (8–10), is dubbed the sub-MIC selective window and spans from the lowest antimicrobial concentration able to select resistance, minimal selective concentration (MSC), to the MIC.

Low antibiotic concentrations can be encountered in the clinical framework. Particularly, they can be found in some specific body compartments due to antibiotics pharmacokinetics, poor patient adherence to treatment, prophylactic therapies, or the use of low-quality medicines (11–13). Further, the continuous anthropogenic release of antibiotics in nature, where they usually remain at low concentrations, has turned this situation into a One-Health issue (14). Soils, sludge, sewage water, rivers, lakes, and even drinking water are drug-polluted nonclinical ecosystems (15, 16). Hence, bacterial pathogens with an environmental origin bring the highest concern, because, from a One-Health perspective, the interconnection between their niches and contiguous ecosystems could result in the spread of resistant bacteria, selected under low drug concentrations in nature, to human host (14).

One of the most relevant pathogens with an environmental origin is Pseudomonas aeruginosa (17), a Gram-negative bacterium able to colonize a wide scope of habitats, given its capacity for adaptation to fluctuating ecosystems (18, 19). It is also one of the most prevalent nosocomial pathogens and causes chronic infections in cystic fibrosis and chronic obstructive pulmonary disease patients (20–22). Its impact on human health cannot be understood without taking into consideration the great assortment of virulence factors it possesses, as well as its intrinsic low susceptibility to antibiotics and ability to acquire higher AR levels (17). The virulence potential of P. aeruginosa is mediated by the production of proteases, toxins, biofilm formation, or motility, among other elements that facilitate infection (23). Concerning acquired AR, the activity of Resistance Nodulation Division (RND)-type multidrug efflux pumps, together with mutations in the drug targets, is particularly relevant (17). One of the antibiotics of choice against infections caused by this pathogen is ciprofloxacin (20, 24).

Ciprofloxacin is a quinolone, a synthetic class of broad-spectrum antibiotics, which has led to it being widely used (25). In fact, ciprofloxacin was the most prescribed quinolone in European countries in 2012 (26, 27). This quinolone inhibits bacterial topoisomerases that are fundamental for DNA replication (28). As a consequence, resistance to this drug can be achieved through mutations in *gyrAB* or *parCE*, the genes that code for a gyrase (type II topoisomerase) and a type IV topoisomerase, respectively (29). Additionally, hyperproduction, normally due to mutations in the genes encoding their regulators, of RND efflux pumps, especially of MexAB-OprM, MexCD-OprJ, MexEF-OprN, and MexXY-OprM, contributes as well to the acquisition of ciprofloxacin resistance by P. aeruginosa (30, 31).

As a result of its wide use, this antimicrobial agent has been found in several natural ecosystems, reaching concentration values as high as 31 μg/mL, 14 μg/mL, 6.5 μg/mL, or 0.2366 μg/mL in two pharmaceutical plant effluents in India and Kenya, an Indian lake, and a hospital effluent in this country, respectively (32–35), among other habitats. Moreover, in clinical settings, there are compartments where ciprofloxacin does not reach bacterial inhibitory concentrations, i.e., inside the sputum of the conductive zones of the airways or on the skin (36, 37). Consequently, investigating the MSCs and the effects of sub-MIC amounts of this quinolone on the emergence and evolution of AR (6, 38–40) takes foremost relevance. Additionally, it should be remarked that overexpression of the genes encoding the above said efflux pumps may alter P. aeruginosa virulence (41–45). Hence, studying the virulence and AR-related phenotypes that are coselected when P. aeruginosa is challenged with sub-MIC amounts of ciprofloxacin is also of interest.

We have previously determined the ciprofloxacin sub-MIC selective window of the P. aeruginosa PA14 model strain (10). However, it remained to be determined if those results may be extrapolated to other P. aeruginosa strains and if a specific ciprofloxacin low concentration could be established as an AR risk threshold for all strains, thereby generalizing our conclusions to the species level. Hence, in the present article, 12 nonduplicate P. aeruginosa clinical isolates, some of them belonging to clades of high clinical concern, were submitted to short-term Adaptive Laboratory Evolution (ALE) assays (46) in the presence of ciprofloxacin, in order to define the width of their sub-MIC selective windows. Further, a risk analysis was performed by determining a fixed ciprofloxacin sub-MIC “risk concentration” that selected resistance in all strains and analyzing its effect on certain bacterial attributes associated with AR and virulence. Knowing that P. aeruginosa environmental and clinical isolates are indiscernible (47) and that the most prevalent clones causing problems at hospitals are also regularly found in natural, nonclinical, ecosystems (48), the information herein included could be a valuable asset to tackle AR from the One-Health angle.

## RESULTS

### Ciprofloxacin sub-MIC selective window of P. aeruginosa clinical isolates depends on their original resistome.

In the current study, we first defined the ciprofloxacin sub-MIC selective windows of 12 P. aeruginosa clinical isolates (see Table 1) and compared them to that of PA14 (10). The strains were chosen looking for the maximum heterogeneity in sequence type (ST), sample origin, and mutational resistome to cover a wide variety. Moreover, high-risk clones with ubiquitous distribution (ST111, ST244) were included in the set (49). All strains were susceptible to ciprofloxacin, according to EUCAST criteria (resistance breakpoint, 0.5 μg/mL), excluding AND04-003 and GAL02-004 (Table 1).

**TABLE 1:** Overview of the 9 days ciprofloxacin sub-MIC selective windows of P. aeruginosa PA14 and 12 clinical isolates^*a*^

Strain (ST)	Sample type	Mutational resistome	MIC (μg/mL)	MS^*b*^ (μg/mL)	Maximum fold-change MIC	Sub-MIC selective concentrations^*c*^	Cross-resistance	Collateral sensitivity
PA14			0.5	0.005	681	1/100 MIC	LEV, CAZ, AMK, ATM,	FOF
						**1/50 MIC**	**LEV**, CAZ, AMK, ATM,	FOF
						**1/25 MIC**	**LEV**	FOF
						**1/10 MIC**	**LEV**, CAZ, AMK,	FOF
						**1/5/MIC**	**LEV**	FOF
						**1/2 MIC**	**LEV**	ATM, FOF
CAN01-002 (111)	Sputum	MexB Q319X, MexY G530S, MexZ Q140K, MexT G276D	0.25	0.005	16	1/50 MIC	LEV, ATM	AMK
						1/25 MIC	LEV, ATM	
						**1/10 MIC**	**LEV**	AMK
						**1/5/MIC**	**LEV**	AMK
						**1/2 MIC**	**LEV**	AMK, IPM
AND04-004A (244)	Tracheal aspirate		0.25	0.005	94	1/50 MIC	**LEV, IPM**	
						**1/25 MIC**	**LEV, IPM**	
						**1/10 MIC**	**LEV, IPM**	AMK
						**1/5/MIC**	**LEV, IPM**	AMK, CAZ, ATM
						**1/2 MIC**	**LEV, IPM**	AMK, ATM
FQSE06-0403 (274)	CF sputum	MexA L338P, MexZ S9P, Mpl S257L, FusA1 Y552C and T671I, FtsI P215L	0.125	0.0125	11	**1/10 MIC**	**LEV**, **AMK**, FOF	
						**1/5/MIC**	**LEV**	
						**1/2 MIC**	**LEV**, FOF	
BAL04-002 (1816)	Blood	MexA K86E, AmpR G295R, AmpC A278G, ParE E215Q	0.4	0.004	85	1/100 MIC	**LEV**, ATM	
						**1/50 MIC**	**LEV**, CAZ, ATM	
						**1/25 MIC**	**LEV**, CAZ, ATM	
						**1/10 MIC**	**LEV**	AMK
						**1/5/MIC**	**LEV**	AMK, CAZ, ATM
						**1/2 MIC**	**LEV**	AMK, cAT, aTM
CAT02-004 (244)	Blood	MexR S88fs, DacB A340G	0.4	0.016	32	**1/25 MIC**	**LEV**, CAZ, AMK	-
						**1/10 MIC**	**LEV**, AMK	-
						**1/5/MIC**	**LEV**, AMK	-
						**1/2 MIC**	**LEV**, AMK	-
GAL02-004 (217)	Sputum	GyrA A908T, GyrB R22C, MexS P225L, Mpl H109Y, AmpC V239A, FusA1 T671A	1	0.1	16	**1/10 MIC**	**LEV**	ATM
						**1/5 MIC**	**LEV**	CAZ, ATM
						**1/2 MIC**	**LEV**	ATM
GAL02-002 (3342)	Sputum	MexZ Y204X, MexS G76S, GalU C242X, OprJ D303V, PmrB A467V	0.125	0.025	4	**1/5/MIC**	**LEV**	AMK
						**1/2 MIC**	**LEV**	AMK, ATM, FOF
FQSE110603 (701)	CF sputum	MexX A38P, MexY N709H and A568T, OprN R363H, AmpDh2 P116S	0.4	0.004	192	**1/100 MIC**	**LEV**, FOF	
						**1/50 MIC**	**LEV**, ATM, FOF	AMK
						**1/25 MIC**	**LEV**, IPM, FOF	AMK
						**1/10 MIC**	**LEV**, ATM, **IPM**, FOF	AMK
						**1/5/MIC**	**LEV**, **IPM**, FOF	AMK
						**1/2 MIC**	**LEV**, **IPM**, FOF	CAZ, AMK
ICA01-004 (698)	Sputum	MexE V156A, AmpR E162Q, ArmZ V266M	0.25	0.01	188	1/25 MIC	**LEV**, CAZ, **ATM**	
						**1/10 MIC**	**LEV**, CAZ, **ATM**, FOF	AMK
						**1/5 MIC**	**LEV**, CAZ, **ATM**, FOF	AMK
						**1/2 MIC**	**LEV**, **IPM**	AMK, ATM
AND04-003 (1227)	Tracheal aspirate	GyrA T83I and K162R, NalD Q97X, MexS L32Q and V333M, MexT R334C	2	0.4	8	**1/5 MIC**	**LEV**, CAZ	
						**1/2 MIC**	**LEV**, CAZ	
CLE03-004 (381)	Tracheal aspirate	MexZ L23fs, AmpC S173N	0.25	0.005	43	1/50 MIC	**LEV**, AMK, ATM, FOF	
						**1/25 MIC**	**LEV**, CAZ, AMK, ATM, FOF	
						**1/10 MIC**	**LEV**, AMK, FOF	
						**1/5 MIC**	**LEV**, FOF	ATM
						**1/2 MIC**	**LEV**	AMK, ATM
FQSE111010 (701)	CF sputum	GyrA Y267N, GyrB R138L, MexX A38P, MexY N709H and A568T, NfxB E75K, OprD Q424E and S403A, OprN R363H, AmpDh2 P116S	0.4	0.04	24	**1/10 MIC**	**LEV**, ATM, FOF	
						**1/5 MIC**	**LEV**, ATM	
						**1/2 MIC**	**LEV**	ATM, FOF

a The table encloses the information about the strains used in this study, their MIC and MSC values to ciprofloxacin, the maximum fold-change of their MICs to this drug (with respect to the one of the parental strains) observed within their 9 days sub-MIC selective windows, and the cross-resistance and collateral sensitivity phenotypes that each concentration within said windows selects for (at least half the replicates must show ≤1/2 or ≥2-fold of the parental strain MIC to be subsumed, as determined using MIC test strips that allow discrimination of small differences in MICs). All clinical isolates had been sequenced and characterized in previous studies (89–91).

b MSCs of ciprofloxacin for all the strains are lower than the highest concentration of this compound found in nature (31 μg/mL) (32).

c Sub-MIC concentrations that selected for at least one population resistant to ciprofloxacin with MIC values ≥EUCAST breakpoint (0.5 μg/mL) are presented in bold. The same applies to the name of the drugs to which at least one population acquired cross-resistance above EUCAST breakpoints. The mutations in the resistomes that are known to be linked to quinolones resistance are underlined (95, 96). The data on PA14 strain come from reference 10. ST, sequence type; CF, cystic fibrosis; fs, frameshift; LEV, levofloxacin; CAZ, ceftazidime; AMK, amikacin; ATM, aztreonam; IPM, imipenem; FOF, fosfomycin.

To determine the width of their sub-MIC selective windows, we undertook short-term ALE assays (9 days) with the 12 isolates, 4 biological replicates for each ciprofloxacin concentration, and 4 control populations grown in the absence of the drug. These are the conditions previously studied with the model strain PA14 (10) and hence allow the comparison of current assays with previous ones. Since P. aeruginosa PA14 presents an MSC of 1/100 of its MIC to ciprofloxacin (10), in these ALEs we used 1/200, 1/100, 1/50, 1/25, 1/10, 1/5, and 1/2 of the isolate MICs to this antimicrobial, in order to cover the potential size of the selective windows. It should be noted here that a sub-MIC was considered part of the selective window when at least half of the biological replicates that evolved under those conditions became resistant at the end of the ALE (≥2-fold of the parental strain MIC value, as determined using MIC test strips that allow discriminating small differences in MICs).

**FIG 1:**
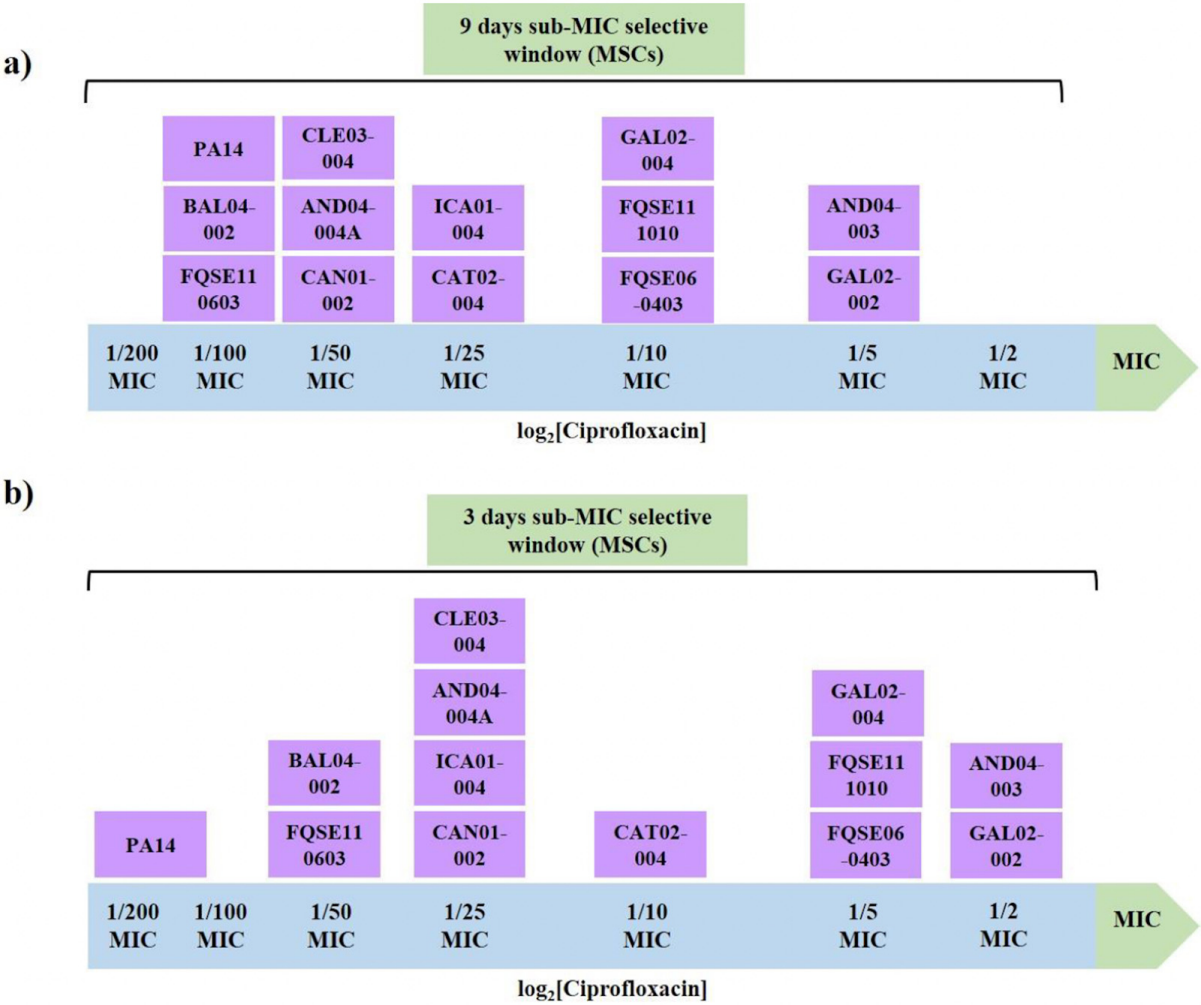
Schematic representation of the ciprofloxacin sub-MIC selective window of P. aeruginosa clinical isolates. These results were obtained by evolving P. aeruginosa populations from 12 clinical isolates under a range of sub-MICs of ciprofloxacin. A sub-MIC was considered as a part of the selective window of an isolate when at least half of the replicates evolved under said concentration became resistant (≥2-fold of the parental MIC value, as determined using MIC test strips) at the end of the ALE. Shown are the data on the windows after 9 days (a) or 3 days (b) of the evolution period. PA14 sub-MIC selective window’s width is incorporated in the figure and comes from reference 10. Information on the strains is comprised in Table 1. MIC values for each replicate and controls are provided in Table S1. MSC, minimal selective concentration.

The results shown in Fig. 1a support that the ciprofloxacin sub-MIC selective window is, at least partly, strain specific, because 5 different windows were observed among the 12 studied clinical isolates. In light of this result, it can be stated that the MSC of ciprofloxacin for a particular strain should not be generalized to all the strains of a bacterial species, at least in the conditions analyzed here. The isolates that exhibited the widest sub-MIC selective window were BAL04-002 and FQSE110603 (1/100 of MIC); conversely, AND04-003 and GAL02-002 were the ones with the narrowest window (1/5 of MIC) (Fig. 1a; Table S1). Broadly speaking, 7 out of 12 clinical isolates presented a large mutational space (MSC ≤1/25 of their MICs), in the range of that previously reported for PA14 (1/100 of its MIC) (10), which should arouse concern for environmental pollution by this quinolone. The differences between the sub-MIC selective window sizes of the different strains might be explained by their different characteristics. Among them, neither the ST (FQSE110603 and FQSE111010 share ST and their MSCs hugely differ) nor the sample type of the clinical isolates seem to be the reasons behind these differences (Table 1). However, we found that a certain connection exists between the observed MSCs, the number of preexistent mutations known to be involved in quinolone resistance (Table 1), the original ciprofloxacin MICs, and the maximum fold change in ciprofloxacin MIC after evolution (Table S1 and Table 1). That is, the majority of isolates that harbored mutations in two or more genes commonly associated with quinolones resistance also displayed higher original MICs to ciprofloxacin, smaller increase in that MIC when facing sub-MIC ciprofloxacin concentrations, and narrower windows and vice versa. For instance, AND04-003 (single nucleotide polymorphisms [SNPs] in *gyrA*, *mexS*, *mexT*, and *nalD*; MIC = 2 μg/mL) presented a MIC increase up to 8-fold and a MSC of 1/5 of MIC, and GAL02-004 (SNPs in *gyrA*, *gyrB*, and *mexS*, MIC = 0.5 μg/mL) presented a MIC increase up to 16-fold and a MSC of 1/10 of MIC. In contrast, AND04-004A (no SNPs in genes encoding AR determinants, MIC = 0.064 μg/mL) showed a MIC increase up to 94-fold and an MSC of 1/50 of MIC, and FQSE110603 (no SNPs potentially involved in quinolones resistance, MIC = 0.125 μg/mL) showed a MIC increase up to 192-fold and an MSC of 1/100 of MIC, supporting the two sides of our hypothesis. These results indicate that mutants already harboring ciprofloxacin resistance mutations, and presenting higher ciprofloxacin MICs, are less likely to acquire new ones at low concentrations of this antibiotic. The reason is that only a limited number of ciprofloxacin resistance mutations can increase the resistance level, or fitness, of preexisting ciprofloxacin-resistant mutants, whereas any ciprofloxacin resistance mutation may do it in isolates originally susceptible to this antibiotic. This may also be the cause of the wider sub-MIC selective window of these strains.

It is worth mentioning that low concentrations of ciprofloxacin broadly increased resistance levels to this drug upon 9-day ALE (up to 192-fold) in a heterogeneous assortment of P. aeruginosa strains, something previously observed (but up to 681-fold) in the PA14 model strain (10). Moreover, the lowest selective concentrations, i.e., the MSCs, may select clinically relevant ciprofloxacin-resistant mutants, presenting MICs above EUCAST clinical breakpoints. This was the case of, for example, FQSE110603 or BAL04-002, which originally presented MICs close to the ones of PA14 and in which 1/100 or 1/50 of MIC (concentrations that can be encountered in several environments), respectively, selected for resistance above said EUCAST breakpoints (Table S1). Therefore, these results buttress the idea that low ciprofloxacin concentrations could select for highly resistant mutants of P. aeruginosa with clinical relevance, regardless of the strain.

### Sub-MICs of ciprofloxacin select for cross-resistance to antibiotics in P. aeruginosa clinical isolates.

**FIG 2:**
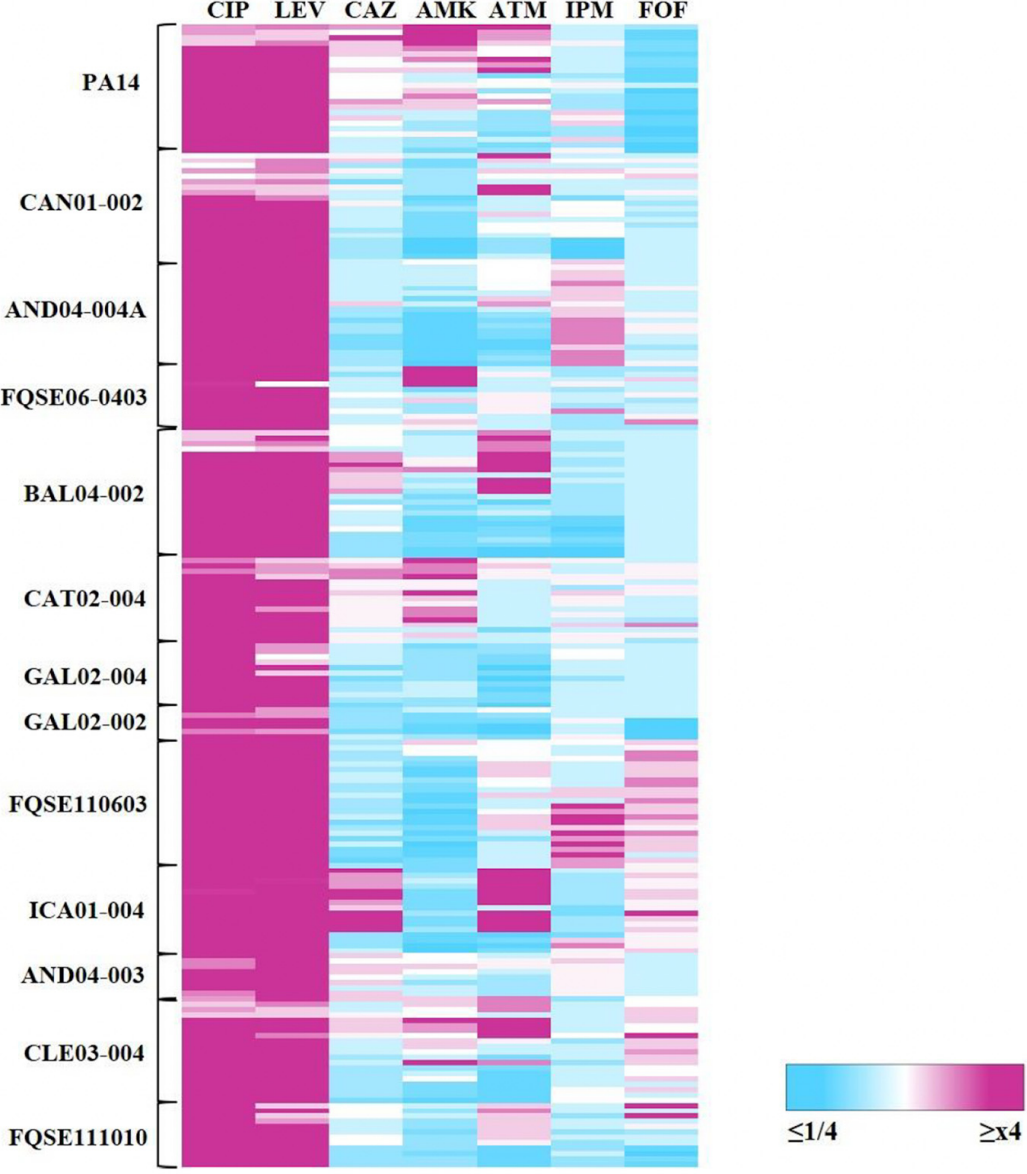
Fold changes of MICs to antibiotics of P. aeruginosa clinical isolate populations evolved under 9 days sub-MIC selective windows of ciprofloxacin. The heatmap represents the fold changes between the MICs of the 9-day evolved populations and their corresponding parental strains. The populations included are the ones which resistance level to ciprofloxacin was ≥2-fold of the parental strain MIC value (as determined using MIC test strips that allow to discriminate small differences in MICs). Said populations of each strain are vertically ordered from the lowest to the highest sub-MIC within their windows. MIC values are encompassed in Table S1 (CIP) and Table S2 (the rest of the drugs). CIP, ciprofloxacin, LEV, levofloxacin; CAZ, ceftazidime; AMK, amikacin; ATM, aztreonam; IPM, imipenem; FOF, fosfomycin.

We have described earlier that P. aeruginosa PA14-resistant mutants, selected under ciprofloxacin sub-MICs, exhibit cross-resistance to other antimicrobial agents (10). To determine if this phenotype could be extrapolated to the 12 clinical isolates here analyzed, we measured the MICs of a set of antibiotics for the 9-day evolved populations, whose resistance level to ciprofloxacin was ≥2-fold of the parental strain MIC (Fig. 2). The chosen antibiotics belong to different structural families, and they are regularly used to treat P. aeruginosa infections (20).

Almost every evolved resistant replicate from all isolates became resistant to other antimicrobials, often belonging to different structural families. Further, the cross-resistance phenotype to the distinct drugs was rather conserved and robust among the biological replicates (Table S2). The most frequent cross-resistance was to levofloxacin, an example of allogenous selection of cross-resistance (50). This is not unexpected, since this antimicrobial is also a quinolone; hence, its mechanisms of action and resistance can be similar to those of ciprofloxacin. We also detected cross-resistance to amikacin, aztreonam, fosfomycin, imipenem, and ceftazidime in several clinical isolates; matching some of these results with the ones of PA14 reference strain (Fig. 2; Table S2). Overall, there is no association between the ciprofloxacin MICs and the strength of the cross-resistance phenotypes, although some of them positively correlated with the concentration of ciprofloxacin. For instance, higher sub-MIC ciprofloxacin concentrations selected for higher cross-resistance to levofloxacin in AND04-004A or BAL04-002 (Fig. 2; Table S2). Thus, our results support that a 9-day evolution of P. aeruginosa clinical isolates in the presence of sub-MICs of ciprofloxacin selects for mutants presenting levels of resistance to the selective drug with no apparent relationship with the levels of cross-resistance to other antimicrobials. Regarding collateral sensitivity, a substantial number of isolates selected within the sub-MIC selective windows presented hypersusceptibility to amikacin, aztreonam, and ceftazidime and in less cases to imipenem and fosfomycin, whereas PA14 acquired a predominant collateral sensitivity to fosfomycin. The latter has been reported in former ALE studies in our laboratory with the PA14 strain in the presence of different antibiotics (10, 51, 52). These studies showed that fosfomycin collateral sensitivity may be caused by the reduction in the expression of *fosa*, which encodes an enzyme that degrades fosfomycin, and of the genes coding for enzymes implicated in the peptidoglycan recycling pathway (53).

Importantly, selection under many sub-MIC ciprofloxacin concentrations frequently gave rise to high levels of cross-resistance that equaled to, or even surpassed, the clinical breakpoints established by EUCAST. This happens in all isolates in the case of levofloxacin cross-resistance, but it is also observed in AND04-004A or FQSE110603 imipenem cross-resistance (Fig. 2; Table S2). In addition, certain clinical isolates were able to gain higher cross-resistance to a specific antibiotic even when they already had an elevated initial MIC (above clinical breakpoint). A paradigmatic example of this phenomenon is FQSE06-0403: despite presenting a MIC to amikacin above EUCAST breakpoints (MIC = 64 μg/mL; breakpoint = 16 μg/mL), probably on account of the aminoglycosides resistance mutations it harbors, in *fusA1* and *mexZ* (54, 55), the populations derived from this clinical isolate that evolved in the presence of 1/10 of MIC to ciprofloxacin presented increased amikacin resistance (MIC ≥256 μg/mL) (Table S2). This result indicates that, even in microorganisms already resistant to an antibiotic, from a clinical perspective, AR may increase, a finding in line with previously published results (52).

In brief, these results aggravate the concern about ciprofloxacin pollution in nature, since, even when present at low concentrations, this antimicrobial can select for clinically relevant MDR P. aeruginosa mutants in a wide range of distinct strains. Hence, from now on, we focus on a more thorough risk analysis in order to unravel the effects of a specific ciprofloxacin sub-MIC on this set of isolates and PA14 strain.

### Defining a sub-MIC threshold for the selection of ciprofloxacin-resistant mutants.

Thus, far, we have centered on defining the ciprofloxacin sub-MIC selective window of a collection of P. aeruginosa clinical isolates using drug concentrations relative to the MIC of each strain. However, from an ecological point of view, it is fundamental to determine a specific ciprofloxacin concentration that selects for antibiotic resistance in all P. aeruginosa clinical isolates tested and to perform a comprehensive risk analysis on P. aeruginosa’s general response to the presence of this concentration. Besides, it is also important to underscore that determining effects of this risk concentration during a shorter lapse than 9-day ALE would be of interest from the risk perspective. The faster AR emerges, the greater the risk. In agreement with the results obtained after 9-day ALE (Fig. 1b; Table S1), we found that 3 days of exposure to ciprofloxacin also selects resistant mutants and that the sub-MIC selective windows are strain specific. By comparing the 3-day to the 9-day interval selection, we observed that every strain’s window broadened its width by one arbitrary concentration, except the one of ICA01-004 (stays the same, 1/25 of its MIC) and PA14 (changes from 1/200 to 1/100 of its MIC). This generalized drop in MSCs over time may suggest that the longer the exposure to low ciprofloxacin concentrations, the higher the chance to select for resistant mutants in even lower amounts of the drug. For its part, the reduction in the window size of the PA14 strain over time could be due to compensatory evolution.

Once established that 3 days of ALE is enough to select resistant mutants, we determined that the lowest ciprofloxacin concentration that selected for resistance to this quinolone, following EUCAST criteria, in all 12 clinical isolates, was 0.04 μg/mL (Table 1; Table S1). From now on, this will be dubbed as risk concentration. Actually, this concentration is lower than many levels of ciprofloxacin contamination that have been reported to date in various ecosystems (32, 34). GAL02-004 and AND04-003 were excluded from this consideration and from further experiments because their original MICs to ciprofloxacin were already above the clinical breakpoint.

We performed 3-day ALE assays using P. aeruginosa PA14 and 10 clinical isolates, in the presence or absence of 0.04 μg/mL of ciprofloxacin, using three biological replicates for each condition. After the ALE, we determined the MICs of the evolved replicates to the selective drug and to antimicrobial agents from other categories, finding that all populations acquired a ciprofloxacin MIC that equaled or surpassed its clinical breakpoint. Besides ciprofloxacin resistance, we found cross-resistance, mainly to levofloxacin and, in certain cases, to imipenem, aztreonam, amikacin, or fosfomycin (Table S3). To get information on mechanisms involved in the acquisition of resistance, we isolated 18 representative ciprofloxacin-resistant single clones from populations of each strain. Their susceptibility to a set of antimicrobials was measured, finding that they also presented cross-resistance phenotypes (Table S4).

### Overproduction of MDR efflux pumps is selected by ciprofloxacin sub-MIC risk concentration.

At this point, we looked for the presence of mutations known to be involved in resistance to quinolones in each of the selected clones. To do that, we screened the presence of genetic changes located in the Quinolone Resistance Determining Regions (QRDRs) of *gyrAB* and *parCE* (quinolone target-encoding genes, corresponding to DNA gyrase and DNA topoisomerase IV, respectively [29]) and in the whole open reading frame of *nfxB*, *mexS*, and *mexT* (encoding regulators of the expression of MexCD-OprJ and MexEF-OprN efflux pumps [56, 57]). The latter were analyzed because recent work in our laboratory stresses the prominent implication of MexCD-OprJ and MexEF-OprN in resistance to ciprofloxacin for many P. aeruginosa strains (10, 58). It is worth pointing out that MexAB-OprM may also contribute to quinolones resistance; however, overexpression of this efflux pump entails ceftazidime and aztreonam cross-resistance (30), and the selected clones did not present this phenotype (Table S4). Therefore, we decided to focus only on the two previously mentioned RND efflux systems. To analyze the presence of genetic changes likely associated with ciprofloxacin resistance, we amplified the aforementioned regions by PCR (primers shown in Table S5) and sequenced the resulting amplicons in the 18 representative clones and their parental strains, using the latter as references.

**FIG 3:**
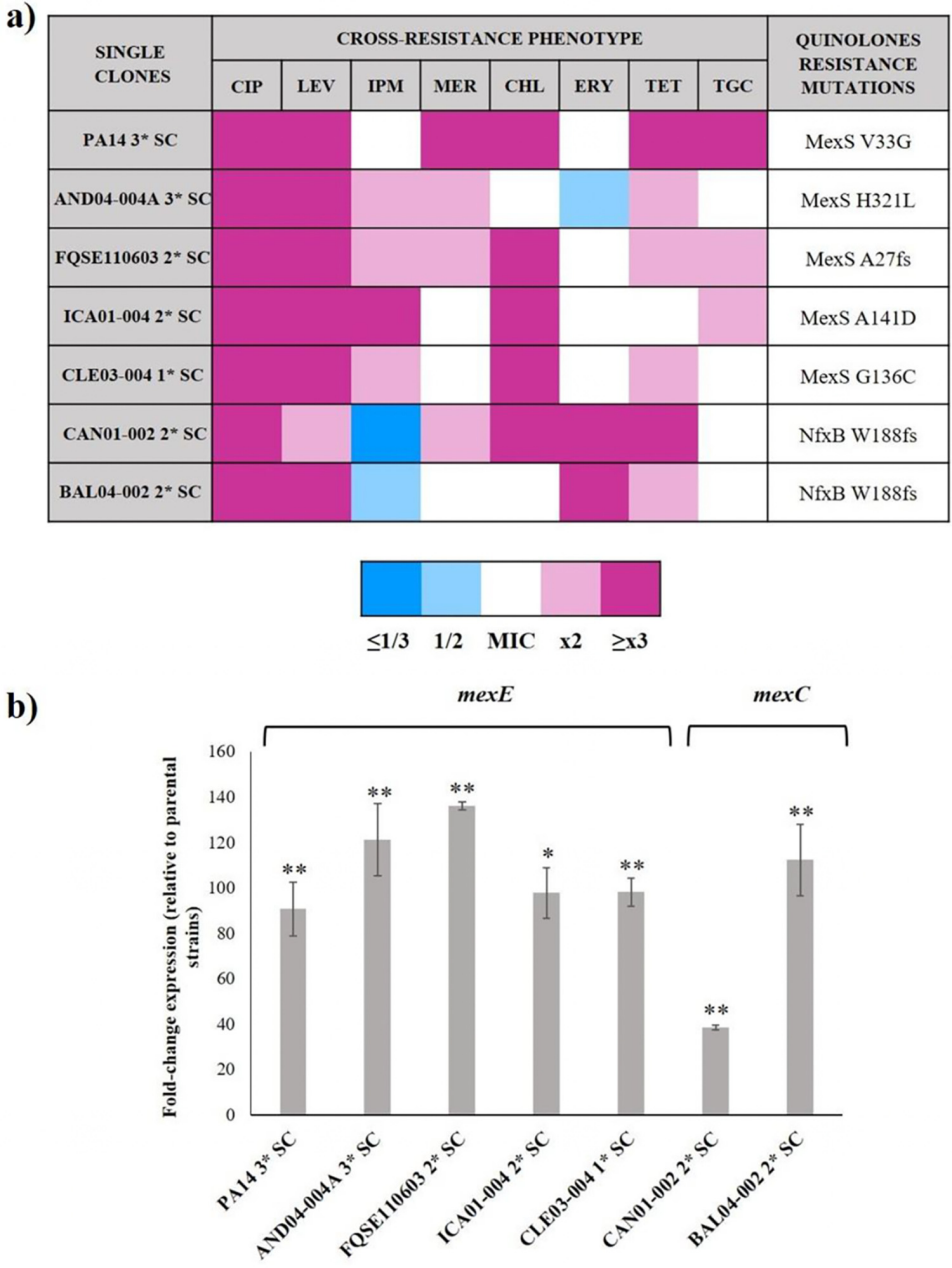
Cross-resistance phenotype and efflux pumps’ implication in P. aeruginosa clones evolved under ciprofloxacin risk concentration. Representative multidrug-resistant clones derived from populations of P. aeruginosa PA14 and 6 clinical isolates evolved under 0.04 μg/mL of ciprofloxacin for 3 days (1*–3* single clone [SC]) were chosen for sequencing genes likely involved in quinolones resistance and measuring their expression level. (a) Cross-resistance phenotype and detected quinolones resistance mutations in the representative clones. MIC values and specific nucleotide changes are enclosed in Table S4. (b) *mexE* and *mexC* expression of the analyzed clones. Fold changes were estimated with respect to the value of each parental strain. As shown, the analyzed clones overexpress either *mexE* or *mexC*. Error bars indicate standard deviations of the results from three independent experiments. Statistically significant differences in the expression level between the analyzed clones and their parental strains were assessed using the Student’s *t* test and are shown (*, *P* < 0.05; **, *P* < 0.005). CIP, ciprofloxacin; LEV, levofloxacin; IPM, imipenem; MER, meropenem; CHL, chloramphenicol; ERY, erythromycin; TET, tetracycline; TGC, tigecycline.

As Fig. 3a and Table S4 show, 7 clones from 5 different isolates had mutations in *mexS*, 4 clones from 2 different isolates harbored mutations in *nfxB* (3 out of 4 shared the same amino acid change), and 2 clones from 2 different isolates selected for SNPs in *gyrA* and *parE*, respectively (both accompanied by a mutation in *mexS*). Interestingly, all analyzed mutants presented SNPs in either *nfxB* or *mexS*, never in both. These genotypes concurred with the results on susceptibility phenotypes: the 7 strains whose derived clones accumulated mutations in the genes encoding an efflux pump regulator exhibited a higher fold change in their MIC to ciprofloxacin and an MDR pattern, whereas the 4 strains whose derived clones had no detectable mutations in the analyzed genes displayed a lower fold change and lacked the aforementioned pattern (Table S4). It cannot be neglected that, as in the case of the sub-MIC selective window’s length, the resistome may play a critical role in this distinction. The clones with no identified SNPs derived from 4 isolates that already harbored mutations in genes encoding efflux pump regulators, which could lead to a preexisting enhanced efflux activity, making the selection of new resistance mutations less imperative (Table 1). Although a detailed analysis of additional mutations potentially involved in the resistance phenotype is not the purpose of the current work, it is worth mentioning that other resistance mutations, located in genes different from the ones here analyzed, might have also been selected by the tested sub-MICs. In any case, these results endorse the contribution of P. aeruginosa MDR efflux pumps as a primary response to ciprofloxacin presence, even at low concentrations.

To further analyze the effect of the mutated regulators on the expression of genes encoding efflux pumps, we analyzed *mexCD-oprJ* and *mexEF-oprN* expression on the batch of representative MDR clones (Fig. 3b). Consistent with their function as transcriptional regulators, all *nfxB* and *mexS* mutants overexpressed the genes coding for MexCD-OprJ and MexEF-OprN efflux pumps, respectively, from 39- to 136-fold of the expression level of the corresponding parental strains. To have a functional validation of the contribution of these pumps to the MDR phenotypes, we determined the MICs of the representative evolved clones in the presence of the efflux pump inhibitor (EPI) phenylalanine-arginine beta-naphthylamide (PAβN). As shown in Table S6, a reduction of the MICs to several antibiotics, namely, ciprofloxacin, levofloxacin, chloramphenicol, erythromycin, tetracycline, and tigecycline, in the presence of the inhibitor, occurs in all cases. We were not able to determine the effect of the EPI on the antibiotic susceptibility of the CAN01-002 2* clone, because this strain cannot grow in the presence of PAβN, a feature in agreement with the previous results (10). Altogether, these data support that the mechanism underlying the MDR of the mutants is the overexpression of MDR efflux pumps, a feature further supported because the substrate range of the upregulated pumps coincided with the specific cross-resistances displayed by the mutants (30, 59) (Fig. 3).

Altogether, these results are rather alarming. Not only 3 days of exposure to ciprofloxacin risk concentration, below registered amounts in nature, selected for resistance to ciprofloxacin in all analyzed P. aeruginosa strains and for MDR in 64% (7 out of 11) of them, but clinically relevant efflux pumps held a prominent role in promoting this phenotype. This information supports the importance of efflux systems in the selection of *de novo* P. aeruginosa-resistant mutants under low concentrations of drugs.

### Ciprofloxacin sub-MIC risk concentration impairs fitness and boosts mutant selection in P. aeruginosa clinical isolates.

**FIG 4:**
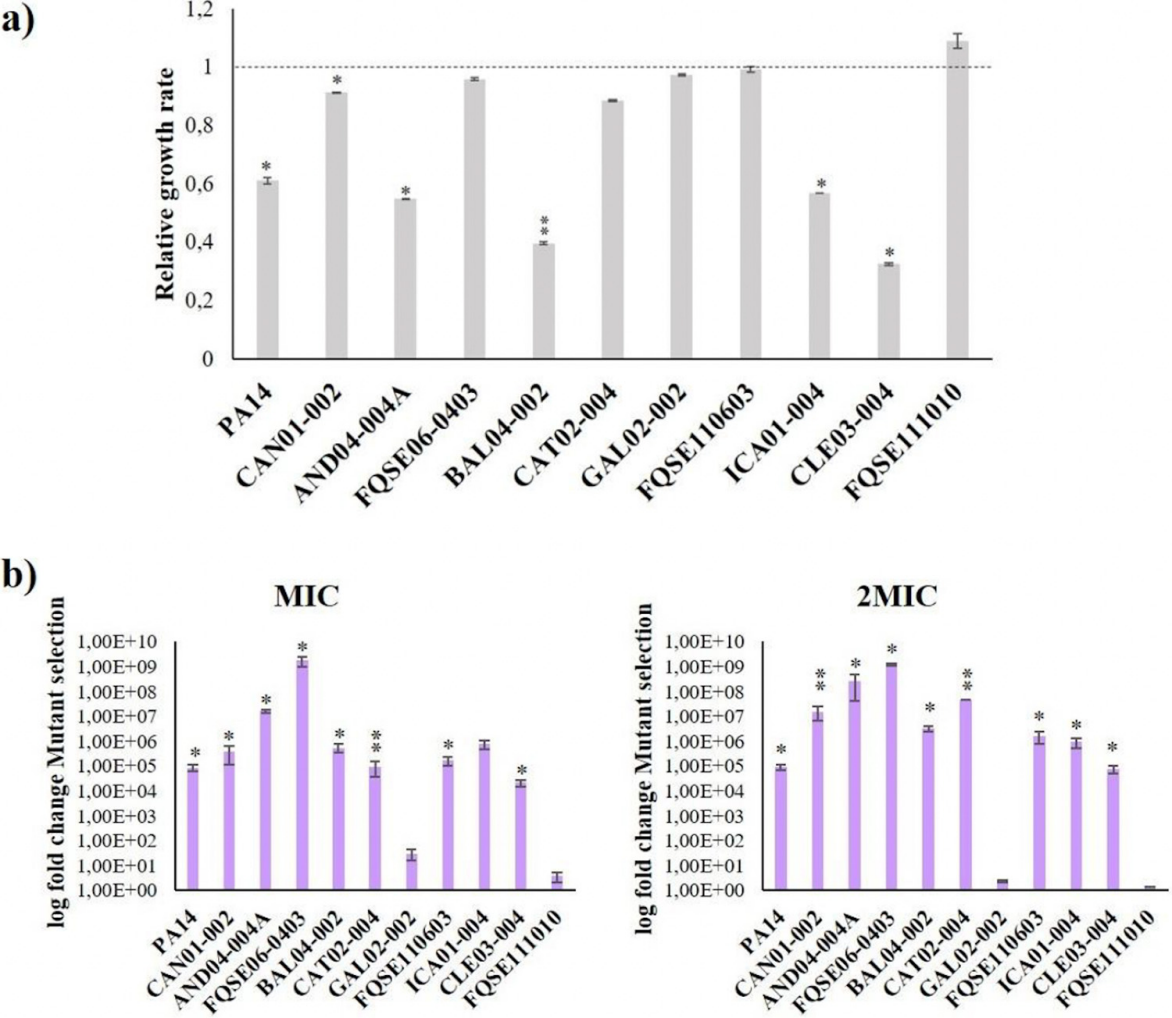
Fitness and mutant selection of P. aeruginosa clinical isolates in the presence of ciprofloxacin risk concentration. (a) Relative fitness of P. aeruginosa PA14 and 10 clinical isolates grown for 30 h in the presence of 0.04 μg/mL of ciprofloxacin, with respect to the ones grown in LBB for the same interval (dotted line). (b) Relative mutant selection of P. aeruginosa PA14 and 10 clinical isolate populations evolved under 0.04 μg/mL of ciprofloxacin for 3 days, with respect to the ones evolved in LBB for the same interval. Mutant selection was uncovered in the presence of MIC and twice the MIC of ciprofloxacin for each strain. In order to calculate fold changes, mutant selection <10^−10^ was considered as 10^−10^. In both assays (a and b), error bars indicate standard deviations from three independent experiments, and statistically significant differences were assessed using the Student’s Student’s *t* test and are shown (*, *P* < 0.05; **, *P* < 0.005).

It has been stated that fitness in the presence of a selecting antimicrobial is a major force driving the selection of antibiotic-resistant mutants at sub-MIC drug concentrations (60). In light of that notion, we measured the growth of the 10 clinical isolates plus PA14 strain, with and without 0.04 μg/mL of ciprofloxacin, in order to decipher whether relative fitness suffered alterations upon quinolone’s presence. As shown in Fig. 4a, 6 out of 11 strains (PA14, CAN01-002, AND04-004A, BAL04-002, ICA01-004, and CLE03-004) presented a significant deficiency in their exponential growth rate when facing ciprofloxacin risk concentration, in comparison with the rate in Luria Bertani broth (LBB), from around 9% (CAN01-002) to almost 70% (CLE03-004). In contrast, no variations were observed among the remaining 5 isolates. That is, the strains with higher fitness costs in the presence of 0.04 μg/mL of ciprofloxacin were the ones that also held higher original susceptibility to ciprofloxacin, as well as the ones with wider sub-MIC selective window, with the exception of FQSE110603 (Table 2).

**TABLE 2:** Overview of the effects of ciprofloxacin risk concentration on P. aeruginosa clinical isolates and its association with their 3-day sub-MIC selective windows^*a*^

			Phenotypic changes upon exposure to 0.04 μg/mL of CIP (fold-changes)								
Strain	Relative CIP MSC (3 days)	CIP mIC of the parental strains	Fitness^*b*^	3-day evolved populations							
				CIP mIC^*c*^	Mutant selection^*c*^		Biofilm formation^*c*^	Elastase activity^*c*^	Pyocyanin production^*c*^	Swarming motility^*c*^	Cross-resistance^*d*^
					MIC	2×MIC					
Narrow sub-MIC selective window											
GAL02-002	1/2 MIC	0.25	1.0	2.4	30	2.3	**1.3**	**0.8**	**1.1**	**2.2**	None
FQSE111010	1/5 MIC	0.25	1.0	1.4	3.6	1.3	1.0	1.0	**1.1**	1.0	None
FQSE06-0403	1/5 MIC	0.19	1.0	5.4	**1.7 × 10^9^**	**1.2 × 10^9^**	**1.7**	1.0	0.9	0.9	LEV, CAZ, AMK, FOF
CAT02-004	1/10MIC	0.25	0.9	2.3	**9.7 × 10^4^**	**4.6 × 10^7^**	0.9	1.2	**0.7**	**4.4**	None
Wide sub-MIC selective window											
CLE03-004	1/25MIC	0.094	**0.3**	13.3	**2.1 × 10^4^**	**7.9 × 10^4^**	1.3	1.0	**0.8**	1.1	LEV, AMK, ATM
ICA01-004	1/25MIC	0.064	**0.6**	13.9	7.7 × 10^5^	**9.3 × 10^5^**	0.9	**0.9**	0.9	**0.3**	LEV, CAZ, AMK, ATM, IPM
AND04-004A	1/25MIC	0.064	**0.5**	10.4	**1.6 × 10^7^**	**2.6 × 10^8^**	1.0	**0.4**	1.0	**0.1**	LEV, IPM
CAN01-002	1/25MIC	0.094	**0.9**	7.8	**3.9 × 10^5^**	**1.6 × 10^7^**	**0.8**	**0.3**	**0.8**	0.9	LEV
BAL04-002	1/50MIC	0.094	**0.4**	10.0	**5.5 × 10^5^**	**3.3 × 10^6^**	1.1	1.0	1.0	0.8	LEV, CAZ, ATM, FOF
FQSE11-0603	1/50MIC	0.125	1.1	13,5	**1.7 × 10^5^**	**1.7 × 10^6^**	1.0	1.1	0.9	1.1	LEV
PA14	1/200MIC	0.064	**0.6**	23.7	**8.6 × 10^4^**	**9.1 × 10^4^**	**0.6**	**0.8**	**0.7**	**0.2**	LEV

a The table summarizes the changes in phenotypic traits of the strains used in this study after 3 days in the presence of ciprofloxacin sub-MIC risk concentration. A statistically significant fold-change in a parameter (excepting CIP MIC fold-change) is highlighted in bold. An MSC of 1/2 of MIC to 1/10 of MIC is dubbed narrow sub-MIC selective window, whereas 1/25 of MIC to 1/200 of MIC is dubbed wide sub-MIC selective window. CIP, ciprofloxacin; LEV, levofloxacin; CAZ, ceftazidime; AMK, amikacin; ATM, aztreonam; IPM, imipenem; FOF, fosfomycin.

b This parameter is measured as the average fold-change of the strain’s fitness in the presence of 0.04 μg/mL of ciprofloxacin for 30 h, with respect to the one in LBB for the same interval.

c This parameter is measured as the average fold-change of the value of populations evolved under 0.04 μg/mL of ciprofloxacin for 3 days, with respect to the ones evolved in LBB for the same interval.

d At least half the replicates must show ≥2-fold of the parental strain MIC to the antibiotic, as determined using MIC test strips that discern small differences, to be subsumed as a cross-resistance.

Another element that may have a primary role in prompting the emergence of AR in sub-MIC conditions is the mutant selection (61). Thus, we tested the relative mutant selection of the P. aeruginosa PA14 and 10 clinical isolate populations evolved in the presence of ciprofloxacin risk concentration for 3 days, in comparison with the ones evolved in LBB for the same period of time. Mutant selection was uncovered in the presence of the MIC and twice the MIC of ciprofloxacin for each strain in LB agar (LBA) plates, thereby providing information on the probability of these isolates to thrive under different amounts of the drug upon which they evolved (Fig. 4b). Partly, the results converged with the ones on fitness: most strains showed a significant increase in the number of mutants that are selected upon exposure to ciprofloxacin sub-MIC risk concentration but especially the ones with wider sub-MIC selective window and larger fitness reduction (i.e., AND04-004A, BAL04-002, or CAN01-002). This also applied inversely, as appreciated in GAL02-002 and FQSE111010, which did not show a significant increase in the number of selected mutants (Table 2). It should be noted here that none of the analyzed strains presented a hypermutator genotype (data not shown). Given these results, low ciprofloxacin concentrations seem to boost the ability of P. aeruginosa to survive under high concentrations of said drug, which strengthens their role in the risk of AR selection.

### Selection by ciprofloxacin sub-MIC risk concentration alters the virulence potential of P. aeruginosa clinical isolates.

It has been described that ciprofloxacin sub-MICs may select for P. aeruginosa mutants with altered virulence and pathogenesis (39, 40, 62). Therefore, we completed the risk analysis by analyzing the virulence phenotype of all isolates evolved under ciprofloxacin sub-MIC risk concentration. Namely, we examined biofilm formation, elastase activity, pyocyanin production, and motility in the PA14 and 10 clinical isolate populations evolved for 3 days in the presence of 0.04 μg/mL of ciprofloxacin, in comparison with the ones grown in LBB for the same period of time.

**FIG 5:**
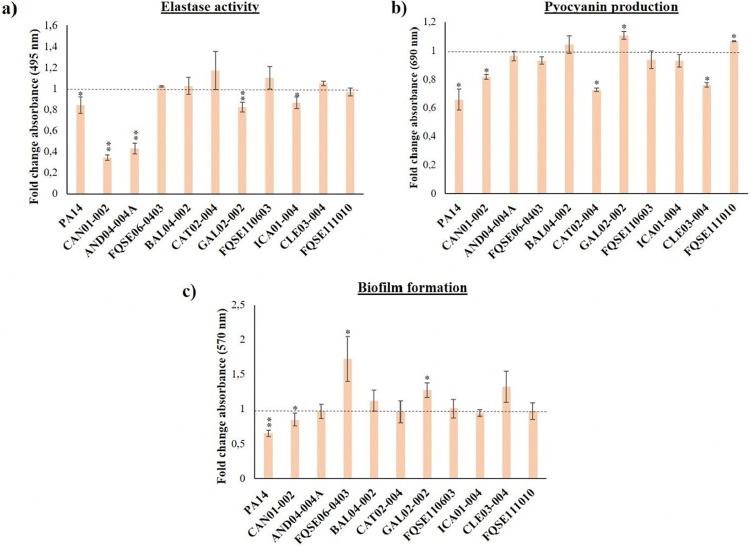
Virulence phenotypes of P. aeruginosa clinical isolates evolved under ciprofloxacin risk concentration. Relative quantification of different phenotypes with relevance for the virulence of P. aeruginosa, measured in P. aeruginosa PA14 and 10 clinical isolate populations evolved under 0.04 μg/mL of ciprofloxacin for 3 days, with respect to the ones evolved in LBB for the same interval (dotted line). The graph presents elastase activity (a), pyocyanin production (b), and biofilm formation (c). Error bars indicate standard deviations from three independent experiments, and statistically significant differences were assessed using the Student’s *t* test and are shown (*, *P* < 0.05; **, *P* < 0.005).

**FIG 6:**
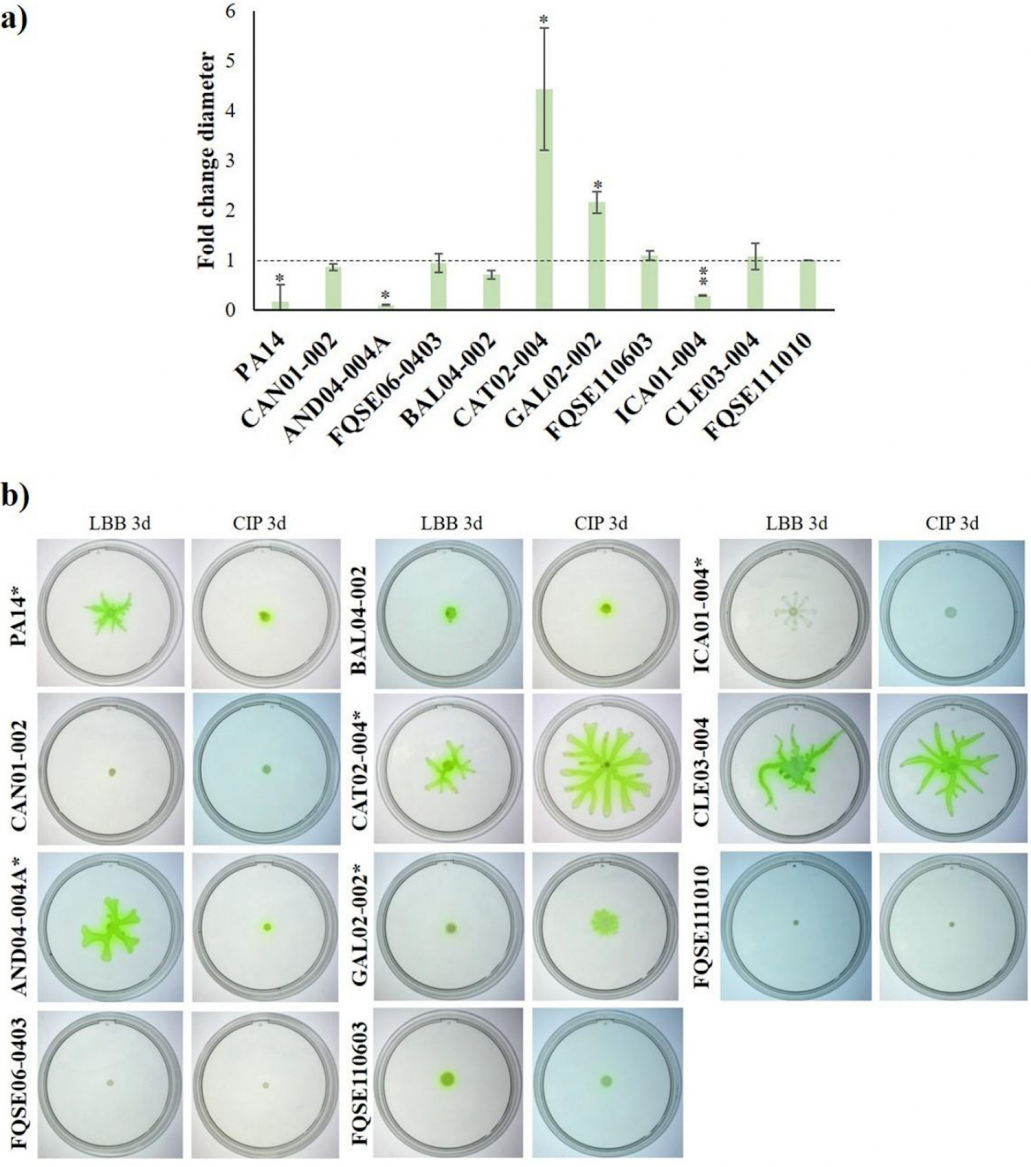
Motility phenotypes of P. aeruginosa clinical isolates evolved under ciprofloxacin risk concentration. (a) The graph depicts the relative diameters of swarming zones in P. aeruginosa PA14 and 10 clinical isolate populations evolved under 0.04 μg/mL of ciprofloxacin for 3 days, with respect to the ones evolved in LBB for the same interval (dotted line). (b) The pictures include the ciprofloxacin-evolved replicates (CIP 3d) and LBB-evolved ones (LBB 3d). Error bars indicate standard deviations from three independent experiments, and statistically significant differences were assessed using the Student’s *t* test and are shown (*, *P* < 0.05; **, *P* < 0.005), both in the graph and the images.

We detected a significant decrease in the elastase activity of PA14, CAN01-002, AND04-004A, GAL02-002, and ICA01-004 populations evolved with ciprofloxacin (Fig. 5a). Regarding pyocyanin, a significant decrease in the production of this toxin was observed in PA14, CAN01-002, CAT02-004, and CLE03-004 populations evolved in the presence of ciprofloxacin, as opposed to what occurred to GAL02-002 and FQSE111010 populations, which presented increased pyocyanin production (Fig. 5b). Referring to biofilm, ciprofloxacin selected for PA14 and CAN01-002 populations with a significant reduction in the amount of this biostructure, while FQSE06-0403 and GAL02-002 populations augmented its formation (Fig. 5c). Lastly, ciprofloxacin selected for mutants presenting a significantly shrunk swarming zone in PA14, AND04-004ª, and ICA01-004 populations, whereas CAT02-004 and GAL02-002 populations showed a broadened swarming area (Fig. 6).

Generally speaking, there is not a robust effect caused by the ciprofloxacin risk concentration on any of the analyzed virulence factors that could be applied to all P. aeruginosa strains. Nonetheless, there seems to exist a certain co-occurrence: most isolates whose virulence declined were the ones with wide sub-MIC selective window, low original MIC to ciprofloxacin, and high increase of ciprofloxacin MIC after ALE, high fitness cost and, on top of that, the ones that selected for efflux pump overproduction when analyzed at single clone level. As a case in point, evolved CAN01-002 populations matched all those criteria, and displayed a significantly lower biofilm formation, elastase activity, and pyocyanin production, after being evolved in the presence of ciprofloxacin risk concentration. On the contrary, ciprofloxacin selected for mutants presenting a heightened virulence phenotype in evolved GAL02-002 populations (regarding biofilm formation, pyocyanin production and swarming motility), as it was precisely the strain with the narrowest sub-MIC selective window, high MIC to ciprofloxacin, and a nonsignificant fitness cost or increase in the emergence of mutants (Table 2). In summary, these results suggest that although a decline in the production of virulence factors is observed in some evolved populations, low ciprofloxacin concentrations might increase the virulence potential of some P. aeruginosa isolates, particularly those with narrower window, (GAL02-002, FQSE111010, FQSE06-0403, and CAT02-004), a feature that magnifies the risk entailed by drug pollution.

## DISCUSSION

Low drug concentrations found in natural ecosystems, released from anthropogenic sources, besides the ones in the clinical context, are a relevant but often neglected menace to human health, because they could drive to the selection of highly resistant bacteria (2). Taking into account that P. aeruginosa is one of the most pervasive human pathogens with an ecological habitat in nature (63), and that ciprofloxacin is one of the antimicrobial compounds that pollutes the most said nature (64), investigating the MSCs of this drug and their effects on various P. aeruginosa strains is of paramount importance. Works about the pleiotropic effects caused by ciprofloxacin sub-MICs have been previously undertaken on this bacterium (6, 39, 40, 62), albeit none have focused on defining the sub-MIC selective windows for different genetic backgrounds or determining a risk concentration to explore ciprofloxacin impact on P. aeruginosa from a One-Health perspective.

Thus, in the current study, we first determined the ciprofloxacin sub-MIC selective windows of 12 P. aeruginosa clinical isolates, finding them to be strain specific and hinged on the mutational resistome. In all cases, extremely low concentrations of this antibiotic enriched bacterial populations in clinically relevant resistant mutants following EUCAST criteria (Tables S1 and S2), some of them presenting an MDR phenotype, as previously observed in the PA14 model strain (10). We do point out that although ciprofloxacin MSCs in P. aeruginosa tend to be low, these concentrations select for high-level resistance. Such a statement is against the classical idea that low-level resistant mutants would prevail at sub-MIC drug conditions (65, 66), although it agrees with more recent research on the subject (3, 38).

Within the conditions used in this work, we have also established the lowest ciprofloxacin concentration threshold above which P. aeruginosa, regardless of the strain, becomes resistant according to EUCAST clinical breakpoint. The danger entailed by this amount (0.04 μg/mL) is not only due to the decreased susceptibility to this quinolone it selects after only 3 days of exposure, but to the selection of mutants overexpressing MDR efflux pumps, via mutations in genes encoding their regulators, rendering an MDR phenotype. The efflux systems that held a conspicuous role in this context were MexEF-OprN and MexCD-OprJ, due to mutations acquired in *mexS* and *nfxB*, respectively (Fig. 3). It is worth mentioning that mutations in these genes, along with the ones located on *gyrA* and *parE*, are frequently found in clinics, being that the overproduction of their associated pumps is one of the main resistance strategies wielded by P. aeruginosa *in vivo* (67–69). In fact, recent work in our laboratory supports a major involvement of MexEF-OprN in P. aeruginosa resistance to ciprofloxacin (58), indicating that this efflux pump may have a leading part in the quinolone resistance arsenal of this pathogen (31, 70). Further, we should not overlook that this type of mutations is expected to be selected for in the presence of above-MIC selective pressure of antibiotics (2, 9); hence, having found them under mild selection, and in such abundance, is both relevant and unsettling.

All these data become more alarming when analyzed through a One-Health prism. The MSCs to ciprofloxacin of the 12 clinical isolates, plus PA14, both a clinical isolate and a model strain, tested in this work, as well as the risk concentration used, which select clinically relevant AR according to EUCAST criteria, are below many of the concentrations levels of this quinolone measured in different ecosystems worldwide (Table 1). For example, high concentrations of ciprofloxacin have been reported in a pharmaceutical plant effluent in India (31 μg/mL) (32), a lake from the same country (6.5 μg/mL) (34), or a wastewater effluent in Kenya (14 μg/mL) (33). In addition, ciprofloxacin is one of the antibiotics that are administered in certain regions within poor-quality medicines that contain substandard quantities of active pharmaceutical compounds (71–73), and its pharmacokinetics are known to render sub-MICs in certain body compartments (36, 37). These data, along with the fact that P. aeruginosa has been found to proliferate in pharmaceutical production environments, which are likely to exhibit the highest levels of drug contamination (74), enlarge the number of situations in which clinically relevant MDR mutants of this pathogen could emerge. As a reminder, there are not P. aeruginosa-specific clones linked to specific habitats; on the contrary, environmental and clinical isolates are indistinguishable (47, 75). This implies that strains infecting a patient can also thrive in nature and vice versa (14). Actually, some of the strains herein studied belong to STs included in the worldwide top 10 high-risk clones of P. aeruginosa (ST111 and ST244) (49), and others are extensively disseminated and associated with AR too (ST274, ST1816 and ST381) (76–78) (Table 1). Moreover, all of them can be found in nonclinical ecosystems (48). Consequently, our results take utmost importance not only from a One-Health point of view but also from a Global-Health angle (14).

To determine the effects of the ciprofloxacin risk concentration on other phenotypes aside from bacterial drug susceptibility, we characterized virulence-related ones (Fig. 5 and 6). Noteworthy, most strains that represented the highest concern from the AR perspective (wide sub-MIC selective window, high fold change in MIC to ciprofloxacin, resistance mutations in single clones) suffered a reduction in their virulence potential (Table 2).

Different works state that low concentrations of ciprofloxacin may select low-virulence P. aeruginosa mutants (6, 39, 40, 79), although some other works state that this issue cannot be generalized (80, 81). Herein we show that the prevalent overexpression of efflux pumps among the resistant evolved isolates might render an impaired virulence of such isolates (Fig. 3). Supporting this conclusion, notwithstanding some exceptions (82), it has been described that MexCD-OprJ and MexEF-OprN upregulation entails a reduction in P. aeruginosa virulence (41, 83, 84), due, at least in part, to the implication of these efflux systems in P. aeruginosa’s quorum sensing response (85, 86). Nevertheless, we are aware that other mutations, not analyzed in the current work, may also contribute to these effects on virulence. For instance, a recent study has proven that the deletion of the flagella-related gene *flgE*, which is involved in swarming motility (87), might alter ciprofloxacin susceptibility and biofilm formation (88). Hence, this kind of noncanonical resistance mutations, if present in the selected mutants, might also have had a role in their reduced virulence. Finally, it should not be disregarded that a few isolates raised their virulence upon ciprofloxacin risk concentration exposure. For instance, CAT02-004, which belongs to the top 10 high-risk clones of P. aeruginosa (ST244), expanded its swarming motility. Therefore, the potential risk to human health caused by ciprofloxacin contamination, even regarding its effect on bacterial virulence, should also be considered.

To summarize, the results of our study provide useful information concerning the threat to global health that ciprofloxacin pollution represents, specifically regarding the high risk of selection of P. aeruginosa MDR mutants with upregulated efflux activity in clinical and nonclinical ecosystems that present low concentrations of this drug.

## MATERIALS AND METHODS

### Bacterial strains, culture conditions, and antibiotic susceptibility assays.

P. aeruginosa clinical strains used in this study were obtained from previously published collections (89–91), and their characteristics are shown in Table 1. Unless otherwise stated, bacteria were grown in Luria Bertani broth (LBB) (Pronadisa) at 37°C with shaking at 250 rpm. MICs of all bacterial populations and single clones in this work (either the ones belonging to the 9-day ALE or the ones from the 3-day ALE) were determined by using MIC test strips (Liofilchem) at 37°C in Mueller-Hinton agar (Pronadisa) plates, with overnight incubation. When required, MICs in the presence of 25 μg/mL of PAβN (Merck) were also determined. The antibiotics analyzed in this work were ciprofloxacin, levofloxacin, ceftazidime, amikacin, aztreonam, imipenem, meropenem, fosfomycin, chloramphenicol, erythromycin, tetracycline, and tigecycline.

### Short-term ALE assays.

Two different ALE assays were conducted in this work. The first one mirrored the characteristics of the one made in (10). 384 bacterial populations from glycerol stock cultures of 12 P. aeruginosa clinical isolates were submitted to a 9-day ALE in the presence of sub-MICs of ciprofloxacin, ranging from 1 to 200 to 1 to 2 of MIC, in glass tubes, of every parental strain (bacterial strains and their MICs are shown in Table 1). Four biological replicates of each genetic background were subjected to evolution in the presence of said drug concentrations or without antibiotic (controls). Each day, the cultures were diluted (1/100), adding 10 μL of bacteria to 1 mL of fresh LBB in test tubes, either harboring the corresponding ciprofloxacin sub-MIC drug concentration or in the absence of antibiotic. Every replicate population was preserved at −80°C after 3 and 9 days of evolution. In the second ALE, 66 bacterial populations from stock cultures of P. aeruginosa PA14 and 10 clinical isolates previously used in the 9-day ALE assays, were submitted to a 3-day experimental evolution in the presence of 0.04 μg/mL of ciprofloxacin. Three biological replicates were subjected to ALE in the presence of said drug or without antibiotic (controls). Conditions of evolution and of storage at −80°C were identical to the ones of the first ALE. From the 3-day evolved populations of this assay, single clones were isolated for further experiments, by streaking them in LB agar (LBA) plates.

### DNA amplification and Sanger sequencing.

Mutations in the QRDRs of *gyrA*, *gyrB*, *parC*, and *parE* and in the whole open reading frames of *nfxB*, *mexS*, and *mexT* were searched in 18 single clones derived from populations of P. aeruginosa PA14 and 10 clinical isolates, as well as in their parental strains. To do so, 7 pairs of primers, which amplified those areas (378 bp of *gyrA*, 511 bp of *gyrB*, 304 bp of *parC*, 592 bp of *parE*, 653 pb of *nfxB*, 1,076 pb of *mexS*, and 1,020 pb of *mexT*), were designed using Primer3 Input software (Table S5) and the available P. aeruginosa PA14 genome sequence. The PCR products were separated by electrophoresis on a 1% agarose (Pronadisa) gel, purified with a QIAquick PCR purification kit (Qiagen), and Sanger sequenced at Macrogen. The sequences were analyzed using SnapGene software.

### RNA extraction and real-time RT-PCR.

Representative MDR evolved single clones from P. aeruginosa PA14 and 6 clinical isolates, along with their parental strains, were grown overnight in LBB at 37°C and 250 rpm. These cultures were inoculated in new flasks at optical density at 600 nm (OD_600_) of 0.01 and incubated until exponential phase (OD_600_ = 0.6) was reached. Then, centrifugation of 10 mL of each of these samples (7,000 rpm, 15 min, 4°C) was performed, and pellets were immediately stored at −80°C. Afterward, RNA was extracted using the RNeasy minikit (Qiagen), following the manufacturer’s protocol. To remove any residual DNA from the samples, two different DNase treatments were made: first with DNase I (Qiagen) and second with TURBO DNase (Ambion). The absence of DNA contamination was checked by PCR using *rplU* primers (Table S5). After, cDNAs were obtained from 5 mg of RNA, using a High-Capacity cDNA Reverse transcription kit (Applied Biosystems).

Real-time RT-PCR was performed using 50 ng of cDNA and Power SYBR green (Applied Biosystems) in an ABI PRISM 7300 real-time PCR system (Applied Biosystems). The primers, designed with Primer3 Input software, were used at 10 μM (Table S5). Since the samples belonged to different genetic backgrounds, the efficiency and specificity of each pair of primers, designed using P. aeruginosa PA14 genome sequence as a reference, were verified using serial dilutions of a cDNA sample. Gene expression data were normalized using the housekeeping gene *rplU*, and the relative amount of mRNA was quantified following the 2^−ΔΔCt^ method (92).

### Bacterial fitness measurement.

Growth curves of 3 independent replicates from 11 strains (P. aeruginosa PA14 plus 10 clinical isolates) were performed. A 10-μL sample of each culture was inoculated in 140 μL of LBB, with or without 0.04 μg/mL of ciprofloxacin, to a final OD_600_ of 0.01, in a 96-well plate (Nunclon Delta Surface; ThermoFisher Scientific). Growth (OD_600_) was monitored every 10 min using a Spark 10M plate reader (TECAN) for 30 h at 37°C. A 20-s shaking was performed before each measurement. Maximum growth rates were calculated resorting to the OD_600_ values at the exponential growth phase. Relative growth rates were obtained by dividing the values of each strain grown in the presence of 0.04 μg/mL of ciprofloxacin by those from the same strain grown in the absence of antibiotics.

### Mutant selection determination.

Mutant selection was determined as described in reference 53, with some modifications. First, ciprofloxacin MICs for the 11 studied strains (P. aeruginosa PA14 plus 10 clinical isolates) were determined, by seeding 10^5^ CFU of every culture in LBA plates containing a range of ciprofloxacin concentrations, choosing the one in which no colonies were observed after overnight incubation at 37°C. Next, all strains evolved under 0.04 μg/mL of ciprofloxacin and in the absence of selective pressure for 3 days were plated in LBA and LBA containing their MIC and twice their MIC. A 100-μL inoculum (OD_600_ = 4) was used, and sequential 1/10 dilutions were performed. After overnight incubation at 37°C, the CFU were counted, and mutant selection was ascertained as the ratio between CFU on ciprofloxacin-containing plates and total CFU. Three replicates of each growth condition and strain were included in the experiment.

### Biofilm formation.

Biofilm formation was tested as described (93), using 96-well microtiter plates (Falcon 3911 Microtest III flexible assay plate) previously sterilized with UV light for 15 min. Briefly, 1:100 dilutions of overnight cultures from all strains were inoculated into microtiter plates (100 μL/well). The plates were incubated at 37°C for 48 h. Subsequently, 25 μL of a 0.1% crystal violet solution in ethanol was added to each well for 5 min. After staining, each well was exhaustively washed with distilled water (4 times). Then, 150 μL of Triton X-100 (0.25%) was added to each well to detach the biofilm, and 100 μL from each sample was transferred to a 96-well microtiter plate (Nunclon Delta Surface; ThermoFisher Scientific) to quantify the biofilm by measuring the OD_570_ in a Spark 10M plate reader (TECAN). Eight replicates of each strain were included in the experiment.

### Pyocyanin and elastase production.

Bacterial samples were grown overnight in 10 mL of LBB at 37°C. After incubation, 1-mL samples were collected and spun down by centrifugation (7,000 rpm, 10 min), and the supernatants were filtered through 0.2-μm pore-size filters (Whatman). Pyocyanin production was determined by placing 100 μL of the filtered supernatants in a 96-well microtiter plate (Nunclon Delta Surface; ThermoFisher Scientific) and measuring the OD_690_ in a Spark 10M plate reader (TECAN). Three replicates of each strain were used in this experiment. The elastase assay was based on the protocol described in (94). One milliliter of Congo red elastin (Sigma-Aldrich) in 100 mM (pH 7.5) Tris-HCl and 1 mM CaCl were added to 100 μL of each filtered supernatant, and the mixture was incubated at 37°C and 250 rpm, for 2 h. Next, samples were centrifuged for 10 min at 13,000 rpm, and the OD_495_ of 100 μL from each strain was determined using the plate reader above described. Three biological replicates, each one with two technical replicates, were included in the assay.

### Motility assay.

Swarming assays were performed in fresh agar plates containing 25 mL of casamino acids medium (0.5% casamino acids, 0.5% filtered glucose, 3.3 mM K_2_HPO_4_, 3 mM MgSO_4_, and 0.5% Bacto-agar). A 4-μL inoculum (OD_600_ = 1) of each strain was placed on the center of the agar plate, and the plates were incubated at 37°C for 18 h. Finally, the motility areas were measured and a picture of every plate was recorded. Three replicates were included in the experiment.
