# Supplementary material for: Low Ciprofloxacin Concentrations Select Multidrug-Resistant Mutants Overproducing Efflux Pumps in Clinical Isolates of Pseudomonas aeruginosa
Source: Microbiol Spectr. 2022 Aug 24;10(5):e00723-22. doi: 10.1128/spectrum.00723-22 (PMC9603996; doi:10.1128/spectrum.00723-22)
Supplement: Supplemental file 1 — Supplemental material. Download spectrum.00723-22-s0001.pdf, PDF file, 0.8 MB [file spectrum.00723-22-s0001.pdf]

Table S1 | MIC values (µg/ml) of *P. aeruginosa* PA14 and 12 clinical isolates after 3 and 9 days of ALE assays under sub-MIC concentrations of ciprofloxacin.

STRAINS	CONCENTRATION (relative to MIC)	REPLICATE	0d	3d	9d
PA14	Without antibiotic	LX1	0,047	0,064	0,064
		LX2	0,047	0,064	0,064
		LX3	0,047	0,064	0,064
		LX4	0,047	0,064	0,064
	1/200	X1	0,047	0,064	0,064
		X2	0,047	0,094	0,064
		X3	0,047	0,064	0,064
		X4	0,047	0,094	0,094
	1/100	X5	0,047	0,064/1	0,125
		X6	0,047	0,064	0,125
		X7	0,047	0,064/0,5	0,094
		X8	0,047	0,047/0,38	0,094
	1/50	X9	0,047	0,75	0,75
		X10	0,047	1	0,75
		X11	0,047	0,75	0,75
		X12	0,047	1	0,75
	1/25	X13	0,047	1,5	1
		X14	0,047	1,5	1
		X15	0,047	1	0,75
		X16	0,047	1	1,5
	1/10	X17	0,047	1,5	1,5
		X18	0,047	2	1,5
		X19	0,047	1,5	1
		X20	0,047	1	1,5
	1/5	X21	0,047	1	16
		X22	0,047	1,5	3
		X23	0,047	2	3
		X24	0,047	2	4
	1/2	X25	0,047	1,5	≥32
		X26	0,047	1,5	6
		X27	0,047	2	12
		X28	0,047	1,5	6
	Without antibiotic	LA1	0,094	0,125	0,125
		LA2	0,094	0,125	0,125
		LA3	0,094	0,125	0,125
		LA4	0,094	0,125	0,125

**CAN01-002**	**1/200**	**A1**	0,094	0,094	0,125
		**A2**	0,094	0,094	0,125
		**A3**	0,094	0,094	0,094
		**A4**	0,094	0,125	0,125
	**1/100**	**A5**	0,094	0,125	0,125
		**A6**	0,094	0,094	0,125
		**A7**	0,094	0,094	0,125
		**A8**	0,094	0,094	0,125
	**1/50**	**A9**	0,094	0,094	0,125
		**A10**	0,094	0,125	0,19
		**A11**	0,094	0,094	0,125
		**A12**	0,094	0,094	0,25
	**1/25**	**A13**	0,094	0,38	0,125
		**A14**	0,094	0,38	0,25
		**A15**	0,094	0,38	0,19
		**A16**	0,094	0,19	0,25
	**1/10**	**A17**	0,094	0,19	0,38
		**A18**	0,094	0,5	0,38
		**A19**	0,094	0,25	0,25/1,5
		**A20**	0,094	0,5	0,5
	**1/5**	**A21**	0,094	0,5	0,5
		**A22**	0,094	0,5	0,5
		**A23**	0,094	0,38	0,5
		**A24**	0,094	0,5	0,75
	**1/2**	**A25**	0,094	0,5	0,5
		**A26**	0,094	0,5	0,5
		**A27**	0,094	0,75	0,75
		**A28**	0,094	0,75	0,75
**AND04-004A**	**Without antibiotic**	**LB1**	0,064	0,064	0,094
		**LB2**	0,064	0,064	0,064
		**LB3**	0,064	0,064	0,064
		**LB4**	0,064	0,047	0,064
	**1/200**	**B1**	0,064	0,064	0,064
		**B2**	0,064	0,064	0,064
		**B3**	0,064	0,064	0,094
		**B4**	0,064	0,064	0,064
	**1/100**	**B5**	0,064	0,094	0,064
		**B6**	0,064	0,064	0,094
		**B7**	0,064	0,094	0,125
		**B8**	0,064	0,064	0,064
	**1/50**	**B9**	0,064	0,064	0,047/0,38
		**B10**	0,064	0,064	0,047/0,38

		**B11**	0,064	0,094	0,047/0,38
		**B12**	0,064	0,094	0,047/0,38
	**1/25**	**B13**	0,064	0,75	0,75
		**B14**	0,064	0,75	0,75
		**B15**	0,064	0,75	0,75
		**B16**	0,064	0,5	0,5
	**1/10**	**B17**	0,064	0,75	1
		**B18**	0,064	0,75	6
		**B19**	0,064	0,75	0,75
		**B20**	0,064	0,5	0,5
	**1/5**	**B21**	0,064	0,75	0,5
		**B22**	0,064	0,5	0,75
		**B23**	0,064	0,75	0,75
		**B24**	0,064	0,5	0,75
	**1/2**	**B25**	0,064	1,5	4
		**B26**	0,064	1	3
		**B27**	0,064	0,5/3	4
		**B28**	0,064	1	1,5
**FQSE06-0403**	**Without antibiotic**	**LC1**	0,19	0,19	0,25
		**LC2**	0,19	0,19	0,19
		**LC3**	0,19	0,19	0,19
		**LC4**	0,19	0,19	0,19
	**1/200**	**C1**	0,19	0,25	0,19
		**C2**	0,19	0,25	0,19
		**C3**	0,19	0,25	0,25
		**C4**	0,19	0,25	0,25
	**1/100**	**C5**	0,19	0,19	0,25
		**C6**	0,19	0,19	0,25
		**C7**	0,19	0,19	0,19
		**C8**	0,19	0,19	0,19
	**1/50**	**C9**	0,19	0,19	0,25
		**C10**	0,19	0,25	0,19
		**C11**	0,19	0,25	0,19
		**C12**	0,19	0,19	0,25
	**1/25**	**C13**	0,19	0,19	0,25
		**C14**	0,19	0,25	0,25
		**C15**	0,19	0,19	0,19
		**C16**	0,19	0,094	0,19
	**1/10**	**C17**	0,19	0,19	0,25/1
		**C18**	0,19	0,19	0,25/1
		**C19**	0,19	0,19	0,25/1
		**C20**	0,19	0,19	0,25/0,75

	**1/5**	**C21**	0,19	1	1,5
		**C22**	0,19	1,5	2
		**C23**	0,19	1	1
		**C24**	0,19	1	1,5
	**1/2**	**C25**	0,19	1,5	1,5
		**C26**	0,19	0,75	1
		**C27**	0,19	0,75	1
		**C28**	0,19	0,5	1,5
**BAL04-002**	**Without antibiotic**	**LD1**	0,094	0,094	0,094
		**LD2**	0,094	0,094	0,094
		**LD3**	0,094	0,094	0,094
		**LD4**	0,094	0,094	0,064
	**1/200**	**D1**	0,094	0,094	0,064
		**D2**	0,094	0,094	0,094
		**D3**	0,094	0,094	0,064
		**D4**	0,094	0,064	0,094
	**1/100**	**D5**	0,094	0,094	0,064/0,19
		**D6**	0,094	0,064	0,064/0,19
		**D7**	0,094	0,064	0,064/0,25
		**D8**	0,094	0,125	0,125
	**1/50**	**D9**	0,094	0,5	0,75
		**D10**	0,094	0,5	0,75
		**D11**	0,094	0,5	0,75
		**D12**	0,094	0,094/0,38	0,5
	**1/25**	**D13**	0,094	0,75	0,75
		**D14**	0,094	1	0,75
		**D15**	0,094	1	0,5
		**D16**	0,094	1	0,75
	**1/10**	**D17**	0,094	1	1
		**D18**	0,094	1	1
		**D19**	0,094	0,75	0,75
		**D20**	0,094	1	0,75
	**1/5**	**D21**	0,094	0,75	1,5
		**D22**	0,094	1,5	1
		**D23**	0,094	1/6	4
		**D24**	0,094	2	2
	**1/2**	**D25**	0,094	6	6
		**D26**	0,094	1,5	8
		**D27**	0,094	1	3
		**D28**	0,094	3	2
	**Without antibiotic**	**LE1**	0,25	0,25	0,19
		**LE2**	0,25	0,25	0,19

		**LE3**	0,25	0,25	0,25
		**LE4**	0,25	0,25	0,19
	**1/200**	**E1**	0,25	0,25	0,38
		**E2**	0,25	0,25	0,38
		**E3**	0,25	0,25	0,38
		**E4**	0,25	0,25	0,38
	**1/100**	**E5**	0,25	0,25	0,38
		**E6**	0,25	0,25	0,25
		**E7**	0,25	0,25	0,38
		**E8**	0,25	0,25	0,38
	**1/50**	**E9**	0,25	0,25	0,38
		**E10**	0,25	0,25	0,25
**CAT02-004**		**E11**	0,25	0,25	0,38
		**E12**	0,25	0,25	0,25
	**1/25**	**E13**	0,25	0,38	0,75
		**E14**	0,25	0,5	1
		**E15**	0,25	0,25	0,75
		**E16**	0,25	0,38	1
	**1/10**	**E17**	0,25	0,75	0,75/4
		**E18**	0,25	1	1
		**E19**	0,25	1	1,5
		**E20**	0,25	1,5	2
	**1/5**	**E21**	0,25	1,5	4/8
		**E22**	0,25	1,5	1
		**E23**	0,25	1,5	2
		**E24**	0,25	1,5	0,5/2
	**1/2**	**E25**	0,25	4	4/8
		**E26**	0,25	1	2
		**E27**	0,25	3	4
		**E28**	0,25	1	3
	**Without antibiotic**	**LF1**	0,5	0,75	0,5
		**LF2**	0,5	0,75	0,75
		**LF3**	0,5	0,75	0,75
		**LF4**	0,5	0,75	0,5
	**1/200**	**F1**	0,5	0,5	0,5
		**F2**	0,5	0,75	0,75
		**F3**	0,5	0,5	0,75
		**F4**	0,5	0,75	0,5
	**1/100**	**F5**	0,5	0,75	0,5
		**F6**	0,5	0,75	0,75
		**F7**	0,5	0,75	0,75
		**F8**	0,5	0,75	0,75

**GAL02-004**	**1/50**	**F9**	0,5	0,5	0,5
		**F10**	0,5	0,5	0,75
		**F11**	0,5	0,5	0,75
		**F12**	0,5	0,75	0,75
	**1/25**	**F13**	0,5	0,75	0,75
		**F14**	0,5	0,75	0,75
		**F15**	0,5	0,75	0,75
		**F16**	0,5	0,75	0,75
	**1/10**	**F17**	0,5	0,75	2
		**F18**	0,5	1,5	3
		**F19**	0,5	0,75	2
		**F20**	0,5	0,75	2
	**1/5**	**F21**	0,5	4	3
		**F22**	0,5	3	2
		**F23**	0,5	4	4
		**F24**	0,5	4	4
	**1/2**	**F25**	0,5	3	4
		**F26**	0,5	4	4
		**F27**	0,5	3	8
		**F28**	0,5	4	4
**GAL02-002**	**Without antibiotic**	**LG1**	0,25	0,25	0,25
		**LG2**	0,25	0,19	0,38
		**LG3**	0,25	0,25	0,25
		**LG4**	0,25	0,38	0,38
	**1/200**	**G1**	0,25	0,19	0,25
		**G2**	0,25	0,25	0,25
		**G3**	0,25	0,25	0,25
		**G4**	0,25	0,25	0,25
	**1/100**	**G5**	0,25	0,25	0,25
		**G6**	0,25	0,25	0,25
		**G7**	0,25	0,38	0,38
		**G8**	0,25	0,38	0,25
	**1/50**	**G9**	0,25	0,25	0,38
		**G10**	0,25	0,25	0,25
		**G11**	0,25	0,19	0,25
		**G12**	0,25	0,38	0,25
	**1/25**	**G13**	0,25	0,25	0,25
		**G14**	0,25	0,25	0,38
		**G15**	0,25	0,19	0,25
		**G16**	0,25	0,19	0,25
		**G17**	0,25	0,38	0,19
		**G18**	0,25	0,38	0,25

	1/10	G19	0,25	0,38	0,25
		G20	0,25	0,38	0,19
	1/5	G21	0,25	0,38	0,38
		G22	0,25	0,5	0,38
		G23	0,25	0,38	1
		G24	0,25	0,5	0,75
	1/2	G25	0,25	1	1
		G26	0,25	1	1
		G27	0,25	1	0,75
		G28	0,25	1	1
FQSE110603	Without antibiotic	LH1	0,125	0,094	0,094
		LH2	0,125	0,094	0,094
		LH3	0,125	0,125	0,094
		LH4	0,125	0,125	0,094
	1/200	H1	0,125	0,125	0,125
		H2	0,125	0,19	0,125
		H3	0,125	0,19	0,125
		H4	0,125	0,125	0,125
	1/100	H5	0,125	0,125	0,5
		H6	0,125	0,25	0,75
		H7	0,125	0,19	1
		H8	0,125	0,19	0,75
	1/50	H9	0,125	0,75	0,75
		H10	0,125	0,5	0,75
		H11	0,125	0,75	0,75
		H12	0,125	0,75	0,75
	1/25	H13	0,125	0,75	0,75
		H14	0,125	1	1
		H15	0,125	1	0,75
		H16	0,125	1,5	0,5
	1/10	H17	0,125	2	1
		H18	0,125	1,5	2
		H19	0,125	1,5	1,5
		H20	0,125	1,5	1,5
	1/5	H21	0,125	1,5	24
		H22	0,125	2	2
		H23	0,125	1,5	2
		H24	0,125	2	2
	1/2	H25	0,125	1	3
		H26	0,125	12	16
		H27	0,125	3	3
		H28	0,125	2	6

ICA01-004	Without antibiotic	LI1	0,064	0,064	0,064
		LI2	0,064	0,064	0,094
		LI3	0,064	0,064	0,094
		LI4	0,064	0,064	0,064
	1/200	I1	0,064	0,064	0,064
		I2	0,064	0,064	0,094
		I3	0,064	0,064	0,064
		I4	0,064	0,064	0,064
	1/100	I5	0,064	0,064	0,064
		I6	0,064	0,064	0,064
		I7	0,064	0,064	0,064
		I8	0,064	0,064	0,064
	1/50	I9	0,064	0,064	0,094
		I10	0,064	0,064	0,094
		I11	0,064	0,064	0,064
		I12	0,064	0,064	0,094
	1/25	I13	0,064	0,5	0,38
		I14	0,064	0,38	0,38
		I15	0,064	0,38	0,38
		I16	0,064	0,38	0,38
	1/10	I17	0,064	0,5	0,25
		I18	0,064	0,38	0,38
		I19	0,064	0,5	0,38
		I20	0,064	0,75	0,75
	1/5	I21	0,064	1	0,5
		I22	0,064	0,75	0,75
		I23	0,064	1	1,5
		I24	0,064	1	0,5
	1/2	I25	0,064	1/6	8
		I26	0,064	2	4
		I27	0,064	1	12
		I28	0,064	2	8
	Without antibiotic	LJ1	2	3	2
		LJ2	2	2	2
		LJ3	2	3	2
		LJ4	2	2	3
	1/200	J1	2	2	2
		J2	2	2	2
		J3	2	2	2
		J4	2	3	2
		J5	2	2	3
		J6	2	2	3

AND04-003	1/100	J7	2	2	2
		J8	2	2	3
	1/50	J9	2	2	2
		J10	2	2	3
		J11	2	2	3
		J12	2	2	2
	1/25	J13	2	3	2
		J14	2	2	2
		J15	2	2	3
		J16	2	2	3
	1/10	J17	2	2	2
		J18	2	3	3
		J19	2	2	2
		J20	2	2	2
	1/5	J21	2	2	8
		J22	2	2	6
		J23	2	3	6
		J24	2	3	8
	1/2	J25	2	12	16
		J26	2	8	8
		J27	2	12	8
		J28	2	8	6
CLE03-004	Without antibiotic	LK1	0,094	0,125	0,094
		LK2	0,094	0,125	0,064
		LK3	0,094	0,094	0,094
		LK4	0,094	0,094	0,094
	1/200	K1	0,094	0,094	0,094
		K2	0,094	0,125	0,064
		K3	0,094	0,125	0,094
		K4	0,094	0,094	0,094
	1/100	K5	0,094	0,125	0,094
		K6	0,094	0,125	0,094
		K7	0,094	0,125	0,125
		K8	0,094	0,094	0,094
	1/50	K9	0,094	0,094	0,25
		K10	0,094	0,125	0,19
		K11	0,094	0,125	0,25
		K12	0,094	0,094	0,19
	1/25	K13	0,094	0,75	0,094/0,38
		K14	0,094	0,75	0,75
		K15	0,094	0,75	0,5
		K16	0,094	0,75	0,38

	**1/10**	**K17**	0,094	1	0,75
		**K18**	0,094	1	1
		**K19**	0,094	1	1
		**K20**	0,094	1	1
	**1/5**	**K21**	0,094	1,5	2
		**K22**	0,094	1,5	1
		**K23**	0,094	1	1,5
		**K24**	0,094	1,5	1
	**1/2**	**K25**	0,094	1,5	2
		**K26**	0,094	1,5	4
		**K27**	0,094	1,5	3
		**K28**	0,094	2	4
**FQSE111010**	**Without antibiotic**	**LL1**	0,25	0,25	0,25
		**LL2**	0,25	0,38	0,38
		**LL3**	0,25	0,25	0,25
		**LL4**	0,25	0,25	0,25
	**1/200**	**L1**	0,25	0,25	0,25
		**L2**	0,25	0,38	0,38
		**L3**	0,25	0,25	0,25
		**L4**	0,25	0,38	0,38
	**1/100**	**L5**	0,25	0,25	0,25
		**L6**	0,25	0,38	0,38
		**L7**	0,25	0,25	0,38
		**L8**	0,25	0,38	0,38
	**1/50**	**L9**	0,25	0,38	0,38
		**L10**	0,25	0,25	0,25
		**L11**	0,25	0,25	0,25
		**L12**	0,25	0,25	0,25
	**1/25**	**L13**	0,25	0,25	0,25
		**L14**	0,25	0,25	0,25
		**L15**	0,25	0,25	0,38
		**L16**	0,25	0,38	0,25
	**1/10**	**L17**	0,25	0,5	1
		**L18**	0,25	0,25	1,5
		**L19**	0,25	0,25	1
		**L20**	0,25	0,5	1,5
	**1/5**	**L21**	0,25	3	2
		**L22**	0,25	3	2
		**L23**	0,25	3	2
		**L24**	0,25	0,38/2	3
	**1/2**	**L25**	0,25	4	4
		**L26**	0,25	2	4

		**L27**	0,25	3	6
		**L28**	0,25	2	4

The table shows the MIC values, at 3 and 9 days, for each replicate population of *P. aeruginosa* PA14 and 12 clinical isolates, evolved under sub-MIC concentrations of ciprofloxacin. Relative sub-MIC concentrations are determined based on the MIC of each strain in LBB. The concentrations belonging to the 9 days sub-MIC selective window are represented as a black cell. All MICs are obtained by E-test strips. Double inhibition halos are shown as two MIC values separated by a slash (X/X), being the highest value the one that was taken into consideration. Ciprofloxacin MIC values ≥ EUCAST breakpoint (0,5 µg/ml) are represented as a grey cell. L(A-L)1-4 refer to control populations evolved in absence of drug (in LBB). The data on PA14 strain come from [1].

Table S2 | MIC values (µg/ml) to antibiotics of different structural families in *P. aeruginosa* PA14 and 12 clinical isolates' populations evolved under 9 days sub-MIC selective windows of ciprofloxacin.

STRAINS	CONCENTRATION (relative to MIC)	REPLICATE	LEV (1)	CAZ (8)	AMK (16)	ATM (16)	IPM (4)	FOF (-)
PA14	Without antibiotic	Parental strain	0,125	0,75	2	1,5	1	64
		LX1	0,19	1	3	2	0,75	48
		LX2	0,19	0,75	3	2	0,75	64
		LX3	0,19	0,75	3	2	0,75	48
		LX4	0,19	1	3	2	1	64
	1/100	X5	0,38	2	8	12	1	48
		X6	0,25	1	12	4	1	24
		X7	0,25	0,75/3	3/8	4	1	24
		X8	0,38	1,5	8	3	1,5	32
	1/50	X9	2	1,5	6	2	1	32
		X10	2	1,5	4	2	1	24
		X11	2	1	6	1/6	1	32
		X12	0,5/2	1	3	1,5/4	1	32
	1/25	X13	2	1,5	4	6	1	24
		X14	3	1	2	0,75	0,75	24
		X15	2	1	2	2	1	24
		X16	3	1	3	2	1,5	64
	1/10	X17	3	1	4	1	1	16
		X18	4	1	6	2	0,75	24
		X19	4	2	4	1,5/4	0,75	24
		X20	4	1,5	4	2	0,75	24
	1/5	X21	24	0,75	2	1	2	16
		X22	6	1,5	2	1	1,5	24
		X23	12	1	1,5	1	2	24
		X24	12	0,75	1,5	1	0,75	16
	1/2	X25	≥32	1	3	0,75	0,75	8
		X26	≥32	0,75	1,5	1,5	2	24
		X27	≥32	0,75	1,5	0,75	0,75	16
		X28	≥32	0,75	1	1	1,5	16
	Without antibiotic	Parental strain	0,25	0,5	4	0,19	12	4
		LA1	0,38	0,75	1,5	0,25	16	12
		LA2	0,38	0,75	3	0,38	16	12
		LA3	0,38	0,75	1,5	0,38	16	8
		LA4	0,38	0,5	3	0,25/2	16	12
		A9	0,38	0,75	4	2	12	4

CAN01-002	1/50	**A10**	0,19/0,75	1	2	0,38	16	6
		**A11**	0,75	0,38	2	0,19	12	4
		**A12**	0,75	0,75	3	0,38	24	6
	1/25	**A13**	0,5	0,5	3	0,25	16	8
		**A14**	0,75	0,25	3	0,19	12	4
		**A15**	0,5	0,75	3	0,19/2	12	4
		**A16**	0,5	0,5	3	0,19/1	12	6
	1/10	**A17**	0,75	0,5	1,5	0,19	6	4
		**A18**	1	0,75	2	0,19	16	3
		**A19**	0,38/4	0,5	3	0,19	16	4
		**A20**	1,5	0,5	2	0,38	16	3
	1/5	**A21**	1,5	0,5	2	0,19	12	4
		**A22**	1,5	0,5	2	0,25	16	3
		**A23**	1,5	0,38	2	0,25	16	4
		**A24**	1,5	0,5	2	0,19	16	4
	1/2	**A25**	1,5	0,38	1	0,125	1,5	4
		**A26**	1,5	0,38	1	0,125	1,5	4
		**A27**	2	0,38	1	0,125	1,5	4
		**A28**	1,5	0,38	1,5	0,19	2	4
AND04-004A	Without antibiotic	**Parental strain**	0,25	1	3	3	2	256
		**LB1**	0,38	1	3	3	3	384
		**LB2**	0,25	1	3	3	3	384
		**LB3**	0,38	1	3	4	2	384
		**LB4**	0,38	1	3	4	2	256
	1/50	**B9**	0,19/1,5	1	4	4	2/4	256
		**B10**	0,19/1,5	1	3	4	3	256
		**B11**	0,19/1,5	1	3	4	2/4	256
		**B12**	0,19/1	1	3	4	4	256
	1/25	**B13**	1/3	1	3	4	6	256
		**B14**	3	1	2	4	4	384
		**B15**	4	1	3	3	4	256
		**B16**	3	1	1,5	2/6	4	256
	1/10	**B17**	2/4	0,75/2	3	8	3	256
		**B18**	16	0,75	1,5	3	4	256
		**B19**	3	0,75	1	2	4	384
		**B20**	3	0,5	1	1,5	6	256
	1/5	**B21**	3	0,75	1	2	6	384
		**B22**	3	0,75	1	1,5	6	384
		**B23**	3	0,5	1	1,5	6	256
		**B24**	3	0,5	1	1	6	256
		**B25**	≥32	0,5	1	1	4	192
		**B26**	24	0,75	1	1,5	6	256

	**1/2**	**B27**	24	0,75	1	2	6	256
		**B28**	6	0,75	0,75	1,5	6	384
**FQSE06-0403**	**Without antibiotic**	**Parental strain**	0,75	0,38	64	0,125	2	64
		**LC1**	0,75	0,25	64	0,125	1,5	96
		**LC2**	0,75	0,25	64	0,094	1,5	96
		**LC3**	0,5	0,25	192	0,125	2	48
		**LC4**	0,75	0,25	96	0,094	1,5	64
	**1/10**	**C17**	1/3	0,38	≥256	0,125	1,5	48
		**C18**	4	0,25	≥256	0,19	1,5	64
		**C19**	1/3	0,38	≥256	0,125	2	128
		**C20**	1	0,38	≥256	0,125	3	64
	**1/5**	**C21**	3	0,38	32	0,125	1,5	64
		**C22**	4	0,5	64	0,19	2	96
		**C23**	3	0,38	128	0,19	2	48
		**C24**	4	0,38	48	0,19	1,5	64
	**1/2**	**C25**	4	0,5	48	0,19	6	64
		**C26**	3	0,38	96	0,125	1,5	96
		**C27**	3	0,38	128	0,125	1,5	192
		**C28**	3	0,5	96	0,125	1,5	48
**BAL04-002**	**Without antibiotic**	**Parental strain**	0,25	0,75	2	2	1	≥1024
		**LD1**	0,25	1	2	3	1	≥1024
		**LD2**	0,25	0,75	2	2	0,5	≥1024
		**LD3**	0,25	0,75	1,5	3	0,75	≥1024
		**LD4**	0,38	1,5	1,5	2	0,75	≥1024
	**1/100**	**D5**	0,19/0,5	1	1,5	2/6	1	≥1024
		**D6**	0,25/1	1	2	2/8	1	≥1024
		**D7**	0,25/0,75	1	2	2/6	0,75	≥1024
		**D8**	0,25/0,5	0,75	2	2/6	0,75	≥1024
	**1/50**	**D9**	2	2	2	8	1	≥1024
		**D10**	1,5	2	3	12	0,75	≥1024
		**D11**	2	3	3	12	0,75	≥1024
		**D12**	2	2	6	8	1	≥1024
	**1/25**	**D13**	3	1,5	2	2	0,75	≥1024
		**D14**	2	1,5	1,5	2/8	1	≥1024
		**D15**	2	1,5	2	1,5/8	0,75	≥1024
		**D16**	2	2	1,5	1/12	0,75	≥1024
	**1/10**	**D17**	2	0,75	1	2	0,75	≥1024
		**D18**	2	0,5	1,5	0,75	0,75	≥1024
		**D19**	2	1	1	1,5	0,75	≥1024
		**D20**	2	0,75	1,5	2	0,75	≥1024
	**1/5**	**D21**	3	0,75	0,75	1	0,38	≥1024
		**D22**	3	0,75	0,75	0,75	0,38	≥1024

		D23	16	1	0,75	1	0,25	≥1024
		D24	4	0,5	1	1	0,38	≥1024
	1/2	D25	16	0,5	1	1,5	0,5	≥1024
		D26	12	0,5	1	1	0,38	≥1024
		D27	4	0,5	0,75	0,5	0,25	≥1024
		D28	6	0,5	0,75	0,5	0,25	≥1024
CAT02-004	Without antibiotic	Parental strain	1,5	2	1	16	2	128
		LE1	2	3	2	16	2	192
		LE2	2	2	1,5	16	1,5	192
		LE3	1,5	3	2	16	2	128
		LE4	1,5	3	3	16	1,5	96
	1/25	E13	3	3	4	24	3	128
		E14	4	4	3	32	2	192
		E15	4	6	3	24	2	192
		E16	3	6	6	24	3	192
	1/10	E17	3/16	3	1,5	16	3	128
		E18	6	3	1,5	16	1,5	192
		E19	6	4	4	16	4	192
		E20	8	3	2	16	3	128
	1/5	E21	≥32	3	1,5	16	3	128
		E22	4	3	3	16	2	128
		E23	12	3	3	16	3	128
		E24	3/12	3	4	16	2	96
	1/2	E25	≥32	3	2	16	4	384
		E26	12	2	1	8	2	128
		E27	24	3	2	16	3	192
		E28	12	3	1	16	3	96
GAL02-004	Without antibiotic	Parental strain	3	1	48	0,75	24	≥1024
		LF1	3	1,5	48	0,75	24	≥1024
		LF2	2	1	48	0,5/4	24	≥1024
		LF3	2	1	48	0,5	24	≥1024
		LF4	2	1	48	0,75	16	≥1024
	1/10	F17	8	1	24	0,5	24	≥1024
		F18	8	1	32	0,5	32	≥1024
		F19	4	1	32	0,38	32	≥1024
		F20	6	1	32	0,38	24	≥1024
	1/5	F21	16	0,5	32	0,19	24	≥1024
		F22	6	1	32	0,38	24	≥1024
		F23	16	0,5	32	0,19	16	≥1024
		F24	16	0,75	48	0,38	24	≥1024
	1/2	F25	12	0,75	48	0,25	24	≥1024
		F26	12	1	48	0,38	24	≥1024

		**F27**	24	0,75	32	0,38	24	≥1024
		**F28**	16	0,5	32	0,19	24	≥1024
**GAL02-002**	**Without antibiotic**	**Parental strain**	0,75	3	32	6	8	384
		**LG1**	0,094/0,75	4	32	6	8	192
		**LG2**	0,125/0,75	2	32	6	8	192
		**LG3**	0,125/0,75	3	24	6	8	192
		**LG4**	0,125/0,75	4	32	6	8	192
	**1/5**	**G23**	2	2	32	1/8	8	384
		**G24**	2	2	16	4	8	384
	**1/2**	**G25**	3	2	16	3	12	32
		**G26**	3	2	12	1	8	48
		**G27**	2	1,5	3/12	0,75	0,125/8	64
		**G28**	4	3	24	3	12	48
**FQSE110603**	**Without antibiotic**	**Parental strain**	0,25	1	6	0,19	1,5	64
		**LH1**	0,38	0,75	8	0,25	2	192
		**LH2**	0,38	1	6	0,25	2	192
		**LH3**	0,38	0,75	6	0,25	1,5	128
		**LH4**	0,38	0,75	6	0,25	1,5	128
	**1/100**	**H5**	2	0,75	12	0,25	2	128
		**H6**	2	1	8	0,25	1,5	96
		**H7**	2	0,75	8	0,25	1,5	192
		**H8**	1,5	0,75	4	0,25	2	192
	**1/50**	**H9**	1,5	1	3	0,38	1,5	128
		**H10**	1,5	0,75	2	0,38	1,5	128
		**H11**	2	1	2	0,38	1,5	128
		**H12**	2	1	4	0,25	1,5	192
	**1/25**	**H13**	1,5	0,75	3	0,25	1,5	192
		**H14**	2/4	0,75	3	0,19	3	128
		**H15**	1,5	1	2	0,38	3	128
		**H16**	1,5	0,75	2	0,19	1,5	192
	**1/10**	**H17**	8	0,75	3	0,19	8	128
		**H18**	8	0,75	1,5	0,25	4	128
		**H19**	6	1	2	0,38	6	192
		**H20**	8	0,5	2	0,38	6	128
	**1/5**	**H21**	≥32	0,75	3	0,38	3	96
		**H22**	8	1	2	0,19	6	192
		**H23**	8	0,5	3	0,125	4	128
		**H24**	12	1	1	0,19	8	128
	**1/2**	**H25**	12	0,5	2	0,19	4	128
		**H26**	≥32	0,5	2	0,19	6	64
		**H27**	12	0,38	3	0,19	4	128
		**H28**	≥32	0,75	3	0,19	4	96

**ICA01-004**	**Without antibiotic**	**Parental strain**	0,38	0,75	8	3	4	32
		**LI1**	0,38	2	4	3	4	32
		**LI2**	0,38	1	4	4	3	32
		**LI3**	0,38	1	4	4	2	32
		**LI4**	0,38	1	4	4	3	32
	**1/25**	**I13**	2	4	4	12	4	48
		**I14**	2	2	6	12	3	64
		**I15**	1,5	2	8	16	3	48
		**I16**	2	2	6	16	3	48
	**1/10**	**I17**	2	4	4	12	3	64
		**I18**	2	4	4	16	3	64
		**I19**	2	2	4	16	3	48
		**I20**	2	1,5	4	3	2	48
	**1/5**	**I21**	2	4	6	16	2	128
		**I22**	3	6	4	16	3	64
		**I23**	8	3	4	24	3	48
		**I24**	2	3	6	24	3	64
	**1/2**	**I25**	16	0,5	3	1	2	48
		**I26**	24	0,5	3	1,5	8	48
		**I27**	≥32	0,5	2	1	12	48
		**I28**	≥32	0,75	2	1,5	6	64
**AND04-003**	**Without antibiotic**	**Parental strain**	6	1,5	3	8	2	≥1024
		**LJ1**	8	2	2	8	3	≥1024
		**LJ2**	8	1,5	3	6	3	≥1024
		**LJ3**	8	2	3	8	3	≥1024
		**LJ4**	12	3	2	8	3	≥1024
	**1/5**	**J21**	24	3	4	8	3	≥1024
		**J22**	≥32	2	4	12	4	≥1024
		**J23**	24	3	6	12	3	≥1024
		**J24**	24	3	4	8	3	≥1024
	**1/2**	**J25**	≥32	2	3	6	3	≥1024
		**J26**	≥32	3	2	6	3	≥1024
		**J27**	≥32	1,5	3	6	3	≥1024
		**J28**	24	3	3	6	3	≥1024
	**Without antibiotic**	**Parental strain**	0,25	1	3	2	0,75	96
		**LK1**	0,25	1	3	1,5	0,75	64
		**LK2**	0,38	1,5	3	3	0,5	64
		**LK3**	0,25	1	3	2	0,75	96
		**LK4**	0,25	0,75	3	2	0,75	128
	**1/50**	**K9**	1	2	6	6	0,5	128
		**K10**	0,75	1	6	2/6	0,75	128
		**K11**	0,5	1	4	2/6	0,75	192

**CLE03-004**		**K12**	0,5	1,5	4	2	0,75	192
	**1/25**	**K13**	1	2	12	8	0,75	192
		**K14**	2	2	8	8	0,75	128
		**K15**	2	2	8	8	0,75	128
		**K16**	0,75	1,5	4	2/8	1	384
	**1/10**	**K17**	1,5	1	6	3	0,5	192
		**K18**	3	1	6	2	0,75	192
		**K19**	3	1	4	2	0,75	256
		**K20**	3	0,75	3	1	0,75	192
	**1/5**	**K21**	1/6	1	3/12	2/6	0,5	192
		**K22**	6	0,75	2	1	0,75	128
		**K23**	4	0,75	3	0,75	0,75	128
		**K24**	4	0,75	4	0,75	0,75	192
	**1/2**	**K25**	6	0,75	1,5	0,75	0,75	96
		**K26**	12	0,75	1,5	0,75	1	192
		**K27**	6	0,75	1,5	0,75	1	96
		**K28**	8	0,5	1	0,75	1	128
**FQSE111010**	**Without antibiotic**	**Parental strain**	0,75	0,75	12	0,25	12	64
		**LL1**	0,75	1	8	0,38	12	64
		**LL2**	1	0,75	12	0,38	8	96
		**LL3**	1	0,75	12	0,25	12	128
		**LL4**	0,75	1	8	0,5	12	64
	**1/10**	**L17**	1,5	1	12	0,5	12	256
		**L18**	4	1	8	0,75	12	64
		**L19**	1,5	1	12	0,5	8	384
		**L20**	2	0,75	8	0,5	12	64
	**1/5**	**L21**	4	0,75	8	0,5	12	96
		**L22**	4	0,75	6	0,5	8	48
		**L23**	4	1	8	0,5	12	64
		**L24**	6	1	8	0,38	8	64
	**1/2**	**L25**	12	0,5	8	0,094	8	24
		**L26**	6	0,5	6	0,094	12	16
		**L27**	16	0,5	8	0,094	8	32
		**L28**	16	0,5	8	0,094	8	24

All MICs are obtained by E-test strips. Double inhibition halos are shown as two MIC values separated by a slash (X/X), being the highest value the one that is taken into consideration. Breakpoints of the antibiotics that are encompassed in EUCAST for *P. aeruginosa* are included in brackets. MIC values ≥ breakpoints are represented as a grey cell. L(A-L)1-4 refer to control populations evolved in absence of drug (in LBB). The

data on PA14 strain come from [1]. LEV: levofloxacin, CAZ: ceftazidime, AMK: amikacin, ATM: aztreonam, IPM: imipenem, FOF: fosfomycin.

Table S3 | MIC values (µg/ml) to antibiotics of different structural families in *P. aeruginosa* PA14 and 10 clinical isolates' populations evolved under 0,04 µg/ml of ciprofloxacin "risk concentration" for 3 days.

STRAINS	CONDITIONS	REPLICATE	ANTIBIOTIC						
			CIP (0,5)	LEV (1)	CAZ (8)	AMK (16)	ATM (16)	IPM (4)	FOF (-)
PA14	Parental strain		0,064	0,19	0,75	1,5	1,5	1	32
	3 days LB	1	0,064	0,19	0,5	2	2	1	16
		2	0,047	0,125	0,75	3	1,5	1	24
		3	0,047	0,19	0,5	2	2	1	24
	3 days CIP	1*	1,5	3	0,75	1,5	2	0,75	24
		2*	1	3	0,75	1	1,5	0,75	16
		3*	1,5	3	1	1,5	1,5	0,75	16
CAN01-002	Parental strain		0,047	0,125	0,5	3	0,19	8	2
	3 days LB	1	0,064	0,19	0,5	2	0,25	12	3
		2	0,064	0,19	0,5	4	0,25	8	3
		3	0,064	0,19	0,75	3	0,25	12	3
	3 days CIP	1*	0,5	1,5	0,5	2	0,094	6	3
		2*	0,75	1	1	3	0,38	8	3
		3*	0,25	0,75	0,5	2	0,19	12	2
AND04-004A	Parental strain		0,064	0,25	0,75	2	3	3	256
	3 days LB	1	0,064	0,25	0,75	3	2	4	192
		2	0,064	0,25	1	2	2	3	256
		3	0,064	0,25	1	2	3	3	384
		1*	0,75	3	0,75	0,75	1,5	6	192

	**3 days CIP**	**2***	0,5	3	0,75	1	2	6	192
		**3***	0,75	2	0,75	0,75	1,5	6	256
**FQSE06-0403**	**Parental strain**		0,125	0,38	0,19	64	0,125	2	12
	**3 days LB**	**1**	0,19	0,38	0,25	64	0,094	1,5	12
		**2**	0,19	0,38	0,38	96	0,125	2	16
		**3**	0,125	0,38	0,38	96	0,125	3	16
	**3 days CIP**	**1***	0,75	2	0,38	192	0,125	1,5	24
		**2***	1	2	0,38	128	0,125	2	24
		**3***	1	2	0,25	128	0,125	2	16
**BAL04-002**	**Parental strain**		0,047	0,25	0,5	3	2	1	192
	**3 days LB**	**1**	0,064	0,25	0,75	2	2	1	192
		**2**	0,047	0,25	0,5	2	2	1	192
		**3**	0,064	0,25	0,38	3	2	1,5	192
	**3 days CIP**	**1***	0,75	2	2	2	12	1,5	512
		**2***	0,5	1,5	2	1,5	12	1,5	512
		**3***	0,5	2	2	1,5	12	2	384
**CAT02-004**	**Parental strain**		0,25	1,5	2	1,5	12	3	96
	**3 days LB**	**1**	0,25	1	2	1	12	3	96
		**2**	0,25	1	2	1	12	3	64
		**3**	0,19	1,5	2	1,5	16	3	96
	**3 days CIP**	**1***	0,5	3	2	1,5	12	3	64
		**2***	0,75	2	2	1	12	4	96
		**3***	0,5	2	3	1,5	12	4	96
	**Parental strain**		0,19	0,5	1,5	24	4	8	48
		**1**	0,125	0,38	2	24	6	6	64

**GAL02-002**	**3 days LB**	**2**	0,125	0,38	3	24	4	6	32
		**3**	0,19	0,5	2	24	6	8	48
	**3 days CIP**	**1***	0,5	0,5	2	24	6	6	48
		**2***	0,5	0,5	2	24	4	8	64
		**3***	0,25	0,38	2	16	6	8	64
**FQSE110603**	**Parental strain**		0,094	0,25	0,75	12	0,25	4	48
	**3 days LB**	**1**	0,094	0,25	0,75	12	0,25	3	64
		**2**	0,064	0,38	0,75	6	0,25	0,5	96
		**3**	0,064	0,38	0,5	8	0,25	1,5	64
	**3 days CIP**	**1***	1	3	0,5	2	0,19	2	48
		**2***	1,5	3	0,19	2	0,25	6	64
		**3***	0,5	2	0,5	3	0,25	3	48
**ICA01-004**	**Parental strain**		0,094	0,25	1	4	3	4	32
	**3 days LB**	**1**	0,094	0,25	1	6	3	4	32
		**2**	0,064	0,25	0,75	6	3	3	24
		**3**	0,094	0,19	1	6	3	4	32
	**3 days CIP**	**1***	1,5	4	4	8	24	8	48
		**2***	1	3	4	12	16	8	24
		**3***	1	3	3	8	16	6	32
**CLE03-004**	**Parental strain**		0,094	0,25	1	6	2	1	64
	**3 days LB**	**1**	0,125	0,25	1	4	3	0,75	128
		**2**	0,125	0,25	0,75	6	3	0,5	128
		**3**	0,125	0,25	1	6	3	0,5	192
	**3 days CIP**	**1***	1,5	4	1,5	4/16	1/8	1	128
		**2***	2	6	1,5	16	1,5/8	1,5	128

		**3***	1,5	4	1,5	8	1,5/12	0,75	128
**FQSE111010**	**Parental strain**		0,25	0,75	0,75	16	0,38	12	48
	**3 days LB**	**1**	0,38	0,75	0,75	12	0,25	12	48
		**2**	0,25	0,75	0,75	16	0,25	12	48
		**3**	0,38	0,75	0,75	12	0,25	16	48
	**3 days CIP**	**1***	0,38	0,75	0,75	16	0,38	8	48
		**2***	0,5	1	1	16	0,38	12	48
		**3***	0,5	1	0,75	16	0,38	12	48

For each strain, 1-3 are the replicate populations evolved in LBB, whereas 1*-3* are the ones evolved in presence of 0,04 µg/ml of ciprofloxacin. All MICs are obtained by E-test strips. Double inhibition halos are shown as two MIC values separated by a slash (X/X), being the highest value the one that is taken into consideration. Breakpoints of the antibiotics that are encompassed in EUCAST for *P. aeruginosa* are included in brackets. MIC values ≥ breakpoints are represented as a grey cell. CIP: ciprofloxacin, LEV: levofloxacin, CAZ: ceftazidime, AMK: amikacin, ATM: aztreonam, IPM: imipenem, FOF: fosfomycin.

Table S4 | MIC values (µg/ml) to antibiotics and mutations in genes involved in quinolones resistance of *P. aeruginosa* single clones from PA14 and 10 clinical isolates' populations evolved under 0,04 µg/ml of ciprofloxacin "risk concentration" for 3 days.

STRAINS	SINGLE CLONES	ANTIBIOTIC											QUINOLONE RESISTANCE MUTATIONS	
		CIP (0,5)	LEV (1)	CAZ (8)	AMK (16)	ATM (16)	IPM (4)	MER (8)	CHL	ERY	TET	TGC	Nucleotide change	Amino acid change
**PA14**	**Parental strain**	0,064	0,19	1	1,5	2	1	0,125	48	48	4	1	-	-
	**1* SC**	1,5	4	1	1	1	1	0,125	≥256	64	12	4	-	-
	**3* SC**	1	2	1	1	1,5	0,75	0,38	≥256	64	12	4	*mexS* T98G	MexS V33G
**CAN01-002**	**Parental strain**	0,094	0,38	1	4	0,19	12	0,38	≥256	96	4	1,5	-	-
	**1* SC**	0,38	1,5	0,38	2	0,094	2	1	96	≥256	3	0,75	*nfxB* 467DelC	NfxB V156fs
	**2* SC**	0,5	1	0,25	1	0,094	1,5	1	≥256	≥256	12	2	*nfxB* A564C	NfxB W188fs
**AND04-004A**	**Parental strain**	0,064	0,25	1	4	3	3	1	≥256	96	6	3	-	-
	**1* SC**	0,38	1,5	0,75	0,75	2	4	1,5	≥256	48	12	3	-	-
	**3* SC**	0,5	2	0,75	1	1,5	8	2	≥256	48	12	3	*gyrA* A346C<br>*mexS* C961T	GyrA N116H<br>MexS H321L
**FQSE06-0403**	**Parental strain**	0,19	0,5	0,38	96	0,125	3	0,19	12	192	6	6	-	-
	**3* SC**	0,5	0,75	0,25	96	0,094	4	0,25	12	≥256	6	6	-	-
**BAL04-002**	**Parental strain**	0,094	0,19	1	3	2	1	0,25	≥256	128	8	3	-	-
	**1* SC**	0,75	1,5	0,75	1,5	0,75	0,5	0,38	≥256	≥256	12	3	*nfxB* T562A	NfxB W188fs
	**2* SC**	4	3	0,75	1,5	0,75	0,38	0,38	≥256	≥256	16	2	*nfxB* A564C	NfxB W188fs
**CAT02-004**	**Parental strain**	0,25	1,5	4	1	12	2	16	≥256	≥256	32	6	-	-
	**2* SC**	0,5	2	3	1	8	2	4	≥256	≥256	24	6	-	-
**GAL02-002**	**Parental strain**	0,19	0,5	1,5	24	4	8	24	96	≥256	4	6	-	-
	**1* SC**	0,5	0,5	2	24	6	6	24	96	≥256	6	6	-	-
**FQSE110603**	**Parental strain**	0,094	0,25	0,75	8	0,25	3	0,047	32	96	3	1,5	-	-
	**1* S**	1,5	4	0,5	1,5	0,125	4	0,064	≥256	96	8	6	*mexS* 548Del 10 bp	MexS R183fs
	**2* SC**	1,5	6	0,5	2	0,19	6	0,125	≥256	64	8	3	*mexS* 80Del 18 bp	MexS A27fs
	**Parental strain**	0,094	0,25	1	4	2	3	0,75	96	96	6	2	-	-

**ICA01-004**	**1* SC**	0,38	1,5	3	4	16	6	1	≥256	128	24	4	-	-
	**2* SC**	1	3	0,75	1,5	1,5	12	1	≥256	128	8	4	*mexS* C422A	MexS A141D
**CLE03-004**	**Parental strain**	0,094	0,25	1	3	2	0,75	0,064	48	192	6	4	-	-
	**1* SC**	1,5	3	0,75	1,5	0,75	1,5	0,094	≥256	192	12	6	*mexS* G406T	MexS G136C
	**2* SC**	0,38	1	1	2	1,5	1	0,094	≥256	192	6	4	*parE* C1103G<br>*mexS* 302InsCGA	ParE A368G<br>MexS G101fs
**FQSE111010**	**Parental strain**	0,25	0,75	1	12	0,38	12	32	≥256	≥256	6	3	-	-
	**3* SC**	0,5	0,75	0,75	12	0,38	8	4	≥256	≥256	4	4	-	-

For each strain, 1*-3* SC are the representative single clones from populations 1*-3* (Table S3) evolved in presence of 0,04 µg/ml of ciprofloxacin for 3 days. All MICs are obtained by E-test strips. Breakpoints of the antibiotics that are encompassed in EUCAST for *P. aeruginosa* are included in brackets. MIC values ≥ breakpoints are represented as a grey cell. The single clones that, based on its MDR phenotype and genotype, were chosen for gene expression analysis, are underlined. Del: deletion, Ins: insertion, Fs: frameshift, bp: base pairs, CIP: ciprofloxacin, LEV: levofloxacin, CAZ: ceftazidime, AMK: amikacin, ATM: aztreonam, IPM: imipenem, MER: meropenem, CHL: chloramphenicol, ERY: erythromycin, TET: tetracycline, TGC: tigecycline.

Table S5 | Oligonucleotides used in this study.

Primer	Sequence (5'-3')	Description
	**PCR amplification**	
gyrA.fw	AGTCCTATCTCGACTACGCGAT	To amplify QRDR* in *gyrA*
gyrA.rv	AGTCGACGGTTTCCTTTTCCAG	
gyrB.fw	CGCGGTGGAACAGGAGATGGGCAAGTAC	To amplify QRDR in *gyrB*
gyrB.rv	CTGGCGGAAGAAGAAGGTCAACAGCAGGGT	
parC.fw	CGAGCAGGCCTATCTGAATTAT	To amplify QRDR in *parC*
parC.rv	GAAGGACTTGGGATCGTCCGGC	
parE.fw	CGGCGTTCGTCTCGGGCGTGGTGAAGGA	To amplify QRDR in *parE*
parE.rv	TCGAGGGCGTAGTAGATTTCCTTGCCGA	
nfxB.fw	TTCTGCACAATGCGCACAA	To amplify the open reading frame of *nfxB*
nfxB.rv	CGTGCCATGCGGCGACGAG	
mexS.fw	TGAGGTTATTCACCCCGTGA	To amplify the open reading frame of *mexS*
mexS.rv	TTCGCTGCGGGTCGTCCCAAT	
mexT.fw	ACGCAAGGCTTGACGGCGA	To amplify the open reading frame of *mexT*
mexT.rv	GCGGTGCGCGATCGATTT	
	**RT-PCR amplification**	
mexC.fw	GACCTGCTGTTCCAGATCG	To amplify *mexC* by RT-PCR
mexC.rv	GTGGCGGTATCGAAGTCCT	
mexE.fw	ACTTCCTCGACAACCAGGTC	To amplify *mexE* by RT-PCR
mexE.rv	GTGCGGTAGACGGTCTTGTT	

rplU.fw	CGCAGTGATTGTTACCGGTG	To check DNA contamination from RNA samples and to amplify the housekeeping gene *rplU* by RT-PCR
rplU.rv	AGGCCTGAATGCCGGTGATC	

*QRDR: Quinolone Resistance Determining Region.

Table S6 | MIC values (µg/ml) to antibiotics of different structural families in presence and absence of the EPI PAβN of single clones from populations of *P. aeruginosa* PA14 and of clinical strains of *P. aeruginosa* evolved under ciprofloxacin "risk concentration".

	CIP		LEV		IPM		MER		CHL		ERY		TET		TGC	
	MH	PAβN	MH	PAβN	MH	PAβN	MH	PAβN	MH	PAβN	MH	PAβN	MH	PAβN	MH	PAβN
**PA14 3* SC**	1	0.19	2	0.094	0.75	0.75	0.38	0.5	≥256	128	64	16	12	2	4	0.75
**AND04-004A 3* SC**	0.5	0.125	3	0.064	4	4	3	2	≥256	≥256	48	24	12	6	4	0.38
**FQSE110603 2* SC**	1.5	0.25	6	1	4	4	0.125	0.125	≥256	≥256	48	24	8	4	3	0.75
**ICA01-004 2* SC**	1	0.25	3	0.5	8	6	0.75	0.75	≥256	≥256	128	32	12	6	4	1
**CLE03-004 1* SC**	1	0.125	4	0.25	0.75	0.75	0.094	0.094	≥256	64	192	12	16	4	8	0.38
**CAN01-002 2* SC**	0.38	X	1	X	1.5	X	0.75	X	256	X	≥256	X	12	X	3	X
**BAL04-002 2* SC**	2	0.25	2	0.125	0.25	0.25	0.38	0.5	≥256	16	≥256	≥256	12	6	4	0.5

For each strain, 1*-3* SC are the representative single clones from populations 1*-3* (Table S3) evolved in presence of 0,04 µg/ml of ciprofloxacin for 3 days. All MICs were obtained by E-test strips, in absence and in presence of 25 µg/ml of the EPI PAβN. "X" indicates the clone that was unable to grow in presence of PAβN.

EPI: efflux pump inhibitor, CIP: ciprofloxacin, LEV: levofloxacin, IPM: imipenem, MER: meropenem, CHL: chloramphenicol, ERY: erythromycin, TET: tetracycline, TGC: tigecycline.

1. Sanz-Garcia, F., S. Hernando-Amado, and J.L. Martinez, *Evolution under low antibiotic concentrations: a risk for the selection of Pseudomonas aeruginosa multidrug-resistant mutants in nature.* Environ Microbiol, 2021.
